# Maternal transmission of SARS‐COV‐2 to the neonate, and possible routes for such transmission: a systematic review and critical analysis

**DOI:** 10.1111/1471-0528.16362

**Published:** 2020-07-22

**Authors:** KF Walker, K O'Donoghue, N Grace, J Dorling, JL Comeau, W Li, JG Thornton

**Affiliations:** ^1^ Division of Child Health, Obstetrics and Gynaecology School of Medicine University of Nottingham Nottingham UK; ^2^ The Irish Centre for Maternal and Child Health Cork University Maternity Hospital University College Cork Cork Ireland; ^3^ School of English University of Nottingham Nottingham UK; ^4^ Department of Pediatrics Faculty of Medicine Dalhousie University Halifax NS Canada; ^5^ Department of Obstetrics and Gynaecology Monash University Clayton Vic. Australia

**Keywords:** artificial feeding, birth, breast‐feeding, caesarean, COVID‐19, disambiguation, duplicate publication, isolation, neonatal infection, pregnancy, SARS‐COV‐2

## Abstract

**Background:**

Early reports of COVID‐19 in pregnancy described management by caesarean, strict isolation of the neonate and formula feeding. Is this practice justified?

**Objective:**

To estimate the risk of the neonate becoming infected with SARS‐CoV‐2 by mode of delivery, type of infant feeding and mother‐infant interaction.

**Search strategy:**

Two biomedical databases were searched between September 2019 and June 2020.

**Selection criteria:**

Case reports or case series of pregnant women with confirmed COVID‐19, where neonatal outcomes were reported.

**Data collection and analysis:**

Data were extracted on mode of delivery, infant infection status, infant feeding and mother–infant interaction. For reported infant infection, a critical analysis was performed to evaluate the likelihood of vertical transmission.

**Main results:**

Forty nine studies included information on mode of delivery and infant infection status for 655 women and 666 neonates. In all, 28/666 (4%) tested positive postnatally. Of babies born vaginally, 8/292 (2.7%) tested positivecompared with 20/374 (5.3%) born by Caesarean. Information on feeding and baby separation were often missing, but of reported breastfed babies 7/148 (4.7%) tested positive compared with 3/56 (5.3%) for reported formula fed ones. Of babies reported as nursed with their mother 4/107 (3.7%) tested positive, compared with 6/46 (13%) for those who were reported as isolated.

**Conclusions:**

Neonatal COVID‐19 infection is uncommon, rarely symptomatic, and the rate of infection is no greater when the baby is born vaginally, breastfed or remains with the mother.

**Tweetable abstract:**

Risk of neonatal infection with COVID‐19 by delivery route, infant feeding and mother‐baby interaction.

## Tweetable abstract

Risk of neonatal infection with COVID‐19 by delivery route, infant feeding and mother‐baby interaction.

This article includes Author Insights, a video abstract available at https://vimeo.com/rcog/authorinsights16362

## Graphical abstract

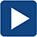

## Introduction

Many early reports of COVID‐19 in pregnancy described management by caesarean, isolation of the neonate from the mother at birth and formula feeding. The reasons included previous experience of the severity of other coronavirus infections in pregnancy as well as an intention to protect the neonate from infection. Of 12 pregnant women with SARS‐CoV in the 2002–2003 pandemic,^1^, ^2^ three mothers died, four women miscarried in the first trimester, two neonates were growth‐restricted and four delivered preterm. Among 11 pregnant women infected with MERS‐CoV,^3^ three mothers and three neonates died. Another factor may have been that the pandemic began in China, where caesarean rates are often over 40% and obstetricians are used to responding to problems by recommending birth by this route.^4^

Expert guidelines have cautiously recommended vaginal birth in the absence of maternal respiratory failure or fetal compromise, as well as breastfeeding with other precautions to minimise maternal to neonate transmission.^5^

Although the number of mothers and neonates included in scientific reports of COVID‐19 pregnancies now number 655 mothers and 666 neonates; many of these reports include the same or overlapping cases.^6^ This may be a particular problem with reports from China. In the City of Wuhan alone, population 12 million, there are 50 hospitals, 19 of which have had cases of COVID‐19 in pregnancy.^7^

The data are complicated by a number of other factors. The mothers involved may have been symptomatic, or asymptomatic, laboratory confirmed or not, and the babies may have been positive or negative on testing, or not tested. The latter group are sometimes assumed to be negative if they were otherwise healthy. Additional complicating factors are different testing modalities available and used across jurisdictions, including RT‐polymerase chain reaction (PCR) or serology, each with its own limitations with respect to sensitivity and specificity.

We have attempted to disentangle duplicate reports. We have used the data extracted to make three comparative estimates for pregnant women with COVID‐19 of the risk of the neonate becoming infected:

1. after vaginal or caesarean birth
2. after breast or formula feeding
3. after rooming‐in with the mother or after mother–baby isolation

Other systematic reviews have been published on this topic.^8^, ^9^, ^10^, ^11^, ^12^, ^13^, ^14^ Our paper is unique in that we have made a concerted effort to report duplicate reports and have critically analysed the risk of neonatal infection by mode of delivery, infant feeding and mother–infant interaction.

## Methods

### Criteria for potentially eligible studies

A protocol for this study was written once data extraction was underway (Appendix S3). Studies were eligible for inclusion if they were case reports or case series of pregnant women with confirmed COVID‐19 infection. There was no language restriction. We only included cases where either the mother had confirmed COVID‐19 based on a positive swab, or there was a high clinical suspicion of COVID‐19 where a swab had not been taken, e.g. symptoms and radiographical evidence in an area of high COVID‐19 prevalence.

### Search strategy

We identified all scientific case reports and case series of confirmed or suspected maternal COVID‐19 in pregnancy. The basis of the list was a curated list kept by the senior author on his personal blog since 22 March 2020 (Appendix S1). The curated list of primary sources is based on a daily PubMed search (Appendix S2) supplemented by alerts from colleagues on social media. After 8 April, this list was supplemented by formal daily searches by KO and KW.

The search was undertaken between 8 April and May 2020 through the following electronic bibliographic databases (MEDLINE, Embase and Maternity and Infant Care Database) and citation tracking of relevant studies. The search terms associated with COVID‐19 used in bibliographic databases were adapted in database‐specific filters. The searches were re‐run just before the final analyses and further studies retrieved for inclusion. The date of the last search was 5 June 2020. The search strategy is shown in Appendix S2.

For assessing cases of possible vertical transmission we attempted to apply the criteria developed by Shah et al.^15^ in order to rank the likelihood of vertical transmission as confirmed, probable, possible, unlikely or not infected. From these we created three tables indicating the rates of baby infection by mode of birth (caesarean or vaginal), rates of infection by breast or formula feeding, and rates by baby rooming‐in or isolation.

### Selection of studies

Titles and abstracts identified by the search strategy were assessed for inclusion by two reviewers (KW, KO). If there was disagreement about whether a report should be included, the full text was obtained for that report.

For all potentially eligible studies, full text copies were sought and independently assessed for inclusion by two reviewers (KW, KO). Disagreements were resolved by discussion; if agreement could not be reached, the study was independently assessed by a third reviewer (JGT).

#### Data extraction and data entry

Data on study quality and content were extracted onto an EXCEL spreadsheet and checked (KW, JGT). Where data was missing, the first author of the paper was contacted by email (*n* = 4). Data was collected on maternal and neonatal outcomes, infant feeding, maternal‐neonatal interaction and for cases with possible vertical transmission, detailed data were collected by virological testing.

#### Study quality

Each included study was judged for the representativeness of the included mothers to three populations of women: all pregnant women with SARS‐CoV2, all pregnant women with COVID‐19 (i.e. symptomatic), all pregnant patients with COVID‐19 admitted to hospital. We also judged the representativeness of the reported babies to the populations of all babies born to women with Covid‐19. The results are shown in Table S2.

### Data analysis

We described the flow of studies through the review (Figure S1), with reasons for being removed or excluded, using PRISMA guidelines.^16^ Characteristics of each study were described and tabulated. No statistical analyses were anticipated.

Patients were not involved in the development of this research and a core outcome set has not been utilised.

## Results

The details of the disambiguation of the reports from Chinese hospitals are shown in Table 1.

**Table 1:** Disambiguation of multiple reports from the same centres in Wuhan City, Hubei Province, China

Hospital name	GRID ref.	Aliases	Studies (Appendix S1 number)	Study analysed (Appendix S1 number)
Zhongnan Hospital of Wuhan University	grid.413247.7	Nil	1, 13, 56, 75	1
Wuhan Children's Hospital	grid.417274.3	Nil	8	8
Maternal and Child Hospital of Hubei Province	grid.440222.2	Hubei Provincial Women and Children's Hospital	5, 17, 30 and 38 (41 women), 44, 54	30
Tongji Hospital, Tongji Medical College, Huazhong University of Science and Technology	grid.412793.a	Nil	2b, 11, 15, 24, 59	15
Union Hospital, Tongji Medical College, Huazhong University of Science and Technology	grid.412839.5	Nil	2a, 2c, 5, 6, 71	2a and 6*
Renmin Hospital of Wuhan University	grid.412632.0	People's Hospital of Wuhan University, Hubei Provincial People's Hospital, First Affiliated Hospital of Wuhan University, Wuhan University Renmin Hospital, Hubei General Hospital	5, 6a, 10, 12, 36, 37, 40	36 and 37**
Central Hospital of Wuhan	grid.440160.7	Nil	39, 73	73

* Data from S2a and S6 are included despite being from the same hospital as follows. S6 reported 10 caesarean births and one vaginal birth, with all babies healthy but no further details. S2a reported three caesarean births of which two were at term, and one preterm. All babies were healthy and all three pharyngeal swabs were negative. We have made the conservative assumption that in total at Union Hospital there were 11 mothers of whom 10 were delivered by caesarean and one vaginally; one of the caesareans was preterm. Of the 11 babies, three, including the preterm one, were negative and eight were not tested.

** Cases from S36 and S37 are included despite being delivered from the same hospital because S36 includes 17 women all delivered by caesarean, and S37 includes three women all of whom were delivered vaginally.

From the list in Appendix S1, we created a database of studies reporting non‐duplicated reports as follows. For studies from Western countries, we judged whether cases were likely to be duplicates by reviewing the hospital and time periods of recruitment. If they overlapped, we excluded the smaller or less informative report as appropriate.

For studies from Wuhan, this was complicated by the issue of translating Chinese names and by some hospitals having multiple English names. We therefore disambiguated centres in the city of Wuhan using the Global Research Identifier Database (GRID) available here www.grid.ac/accessed 1 May 2020). From each report we extracted the English name for the hospital in which the patients had been cared for or delivered and entered this in the GRID ‘disambiguator’ and retrieved the hospital GRID identifier.

One of the referees who had lived in Wuhan felt that there were mistakes in the GRID database. We therefore invited a co‐author WL, who had also worked in Wuhan for some years, to join us. He manually checked the initial GRID centre disambiguation and made corrections. Once this manual check was complete, we grouped all reports which included patients delivered in the same hospital and reported the largest series available with useful information.

For two hospitals, ‘Wuhan Union Hospital’ grid.412839.5 and ‘Renmin Hospital of Wuhan University’ grid.412632.0, we identified two papers where there was internal evidence of non‐overlap from which useful data could be extracted. The details are described in the footnotes to Table 1.

For hospitals in cities other than Wuhan without GRID identifiers we recorded the hospital name as given in the paper and assumed no duplication with Wuhan cases.

If the hospital in which patients were treated was not specified in the report, we attempted to deduce this from the affiliations of the first, last or corresponding author. However, it soon became clear that this method led to ambiguous results and added little to the reported identification. As it was impossible to ascertain whether these hospitals were duplicates, they were excluded.

Following disambiguation, we included 49 studies from China, USA, Europe, Honduras, Korea, Australia, Peru, Canada, UK and Iran. These studies included 666 neonates and 655 women where information was provided on the mode of delivery and the infant's infection status. Ten women in the included studies underwent caesarean birth for twins and one woman had a vaginal birth of both twins.

The risks of neonatal infection after vaginal and caesarean birth are shown in Table 2, of infection after breast or formula feeding or expressed milk in Table 3 and after rooming‐in or separation in Table 4.

**Table 2:** Mode of delivery and neonatal outcomes

	Centre/hospital (study numbers from Appendix S1)	Vaginal					Caesarean				
		Total neonates	Infected	Not infected	Not tested	Died	Total neonates	Infected	Not infected	No test	Died
China	Zhongnan Hospital of Wuhan University (1)	0	0	0	0	0	9	0	6	3	0
	Wuhan Children's Hospital (8)	0	0	0	0	0	3	3	0	0	0
	Maternal and Child Hospital of Hubei Province (30)	2	0	0	2	0	15	0	3	12	0
	Central Hospital of Wuhan (73)	5	0	5	0	0	18	0	18	0	0
	Union Hospital, Tongji Medical College, Huazhong University of Science and Technology (2a and 6)	1	0	0	0	0	13	0	3	0	0
	Tongji Hospital, Tongji Medical College, Huazhong University of Science and Technology (15)	0	0	0	0	0	7	1	2	4	0
	First Affiliated Hospital of Sun Yat‐sen University (3)	3	0	3	0	0	10	0	10	0	1
	Renmin Hospital of Wuhan University (36 and 37)	3	0	3	0	0	17	0	17	0	0
	Affiliated Infectious Hospital of Soochow University, Suzhou. No GRID listing (19)	0	0	0	0	0	1	0	1	0	0
	Beijing YouAn Hospital (34)	1	0	1	0	0	0	0	0	0	0
	No. 2 People's Hospital of Hefei City Affiliated to Anhui Medical University (62)	0	0	0	0	0	1	0	1	0	0
USA	New York Presbyterian Hospital Columbia (27)	10	0	10	0	0	8	0	8	0	0
	MedStar Washington Hospital Center (21)	1	0	1	0	0	0	0	0	0	0
	Good Samaritan Hospital, Ohio (50)	0	0	0	0	0	1	0	1	0	0
	Hospital of the University of Pennsylvania (65)	0	0	0	0	0	3	0	3	0	0
	Washington University in St. Louis, Missouri (69)	0	0	0	0	0	1	0	1	0	0
	New York Winthrop Hospital (91)	0	0	0	0	0	1	0	1	0	0
	New York University, Langone Health (85)	7	0	7	0	0	4	0	4	0	0
	San Francisco (89)	0	0	0	0	0	1	0	1	0	0
	Livingstone, New Jersey (111)	0	0	0	0	0	2	1	1	0	0
	Stanford University Hospital (115)	1	0	1	0	0	0	0	0	0	0
	Weill Cornell Medicine, New York (118)	0	0	0	0	0	1	0	1	0	0
	Beaumont Hospital Dearborn, Michigan (123)	8	0	8	0	0	4	0	4	0	0
	Maimonides Medical Center, Brooklyn (113)	46	0	30	16	0	22	0	18	4	0
Honduras	Hospital Escuela of Tegucigalpa (18)	1	0	1	0	0	0	0	0	0	0
Sweden	Southern General Hospital, Stockholm (20)	0	0	0	0	0	2	0	2	0	0
Korea	Daegu Fatimal Hospital (22)	0	0	0	0	0	1	0	1	0	0
Turkey	Ankara University (31)	0	0	0	0	0	1	0	1	0	0
Italy	IRCCS Fondazione Policlinico Universitario Agostino Gemelli, Rome (76)	0	0	0	0	0	2	1	1	0	0
	Sant'Anna Hospital, Turin (79)	1	1	0	0	0	0	0	0	0	0
	Palma (109)	3	0	2	1	0	1	0	1	0	0
	12 Italian hospitals (117)	34	3	31	0	0	22	1	21	0	0
Portugal	Hospital Pedro Hispano in Porto (105)	4	0	4	0	0	6	0	6	0	0
Australia	Gold Coast University Hospital (45)	1	0	1	0	0	0	0	0	0	0
Canada	Mount Sinai Hospital (48A)	0	0	0	0	0	1	0	0	1	0
	Toronto (103)	0	0	0	0	0	1	1	0	0	0
France	Antoine Béclère Hospital (48B)	0	0	0	0	0	1	0	0	1	0
Spain	Madrid (125)	18	0	18	0	0	5	0	5	0	0
Lima, Peru	British American Hospital (51)	0	0	0	0	0	1	1	0	0	0
India	Designated COVID hospital (58)	0	0	0	0	0	1	0	1	0	0
Iran	Vali‐e‐asr Hospital, Zanjan (43)	1	0	0	1	1	0	0	0	0	0
	Tehran/Rasht/Qom/Zanjan (67)	1	0	0	1	1	7	1	5	1	0
	Imam Khomeini Hospital, Sari (70)	0	0	0	0	0	1	1	0	0	0
	Imam Reza Hospital of Tabriz (101)	0	0	0	0	0	1	0	1	0	0
UK	UKOSS (92)	107	4	102	0	5	161	8	148	0	0
Belgium	Cliniques Universitaires Saint Luc (100)	0	0	0	0	0	1	1	0	0	0
Netherlands	NethOSS (141)	33	0	33	0	0	16	0	16	0	0
Total		292	8	261	21	7	374	20	313	26	1

**Table 3:** Breastfeeding and neonatal outcomes

Centre/hospital (study numbers; Appendix S1)	Breastfed					Formula fed					Not reported					Expressed breast milk				
	Total	Infected	Not infected	No test	Died	Total	Infected	Not infected	No test	Died	Total	Infected	Not infected	No test	Died	Total	Infected	Not infected	No test	Died
**China**																				
Zhongnan Hospital of Wuhan University (1)	0	0	0	0	0	0	0	0	0	0	9	0	6	3	0	0	0	0	0	0
Wuhan Children's Hospital (8)	0	0	0	0	0	0	0	0	0	0	3	3	0	0	0	0	0	0	0	0
Maternal and Child Hospital of Hubei Province (30)	0	0	0	0	0	17	0	3	14	0	0	0	0	0	0	0	0	0	0	0
Central Hospital of Wuhan (73)	0	0	0	0	0	0	0	0	0	0	23	0	23	0	0	0	0	0	0	0
Union Hospital, Tongji Medical College (2a and 6)	0	0	0	0	0	0	0	0	0	0	3	0	3	0	0	0	0	0	0	0
Tongji Hospital, Tongji Medical College (15)	0	0	0	0	0	0	0	0	0	0	7	1	2	4	0	0	0	0	0	0
First Affiliated Hospital of Sun Yat‐sen University (3)	0	0	0	0	0	0	0	0	0	0	13	0	13	0	0	0	0	0	0	0
Renmin Hospital of Wuhan University (36 and 37)	0	0	0	0	0	3	0	3	0	0	17	0	17	0	0	0	0	0	0	0
Affiliated Infectious Hospital of Soochow University, Suzhou (19)	0	0	0	0	0	1	0	1	0	0	0	0	0	0	0	0	0	0	0	0
Beijing YouAn Hospital (34)	0	0	0	0	0	0	0	0	0	0	1	0	1	0	0	0	0	0	0	0
No. 2 People's Hospital of Hefei City Affiliated to Anhui Medical University (62)	0	0	0	0	0	1	0	1	0	0	0	0	0	0	0	0	0	0	0	0
**USA**																				
New York Presbyterian Hospital Columbia (27)	17	0	17	0	0	1	0	1	0	0	0	0	0	0	0	0	0	0	0	0
MedStar Washington Hospital Center (21)	0	0	0	0	0	0	0	0	0	0	0	0	0	0	0	1	0	1	0	0
Good Samaritan Hospital in Cincinnatti, Ohio (50)	0	0	0	0	0	1	0	1	0	0	0	0	0	0	0	0	0	0	0	0
Hospital of the University of Pennsylvania (65)	0	0	0	0	0	0	0	0	0	0	3	0	3	0	0	0	0	0	0	0
Washington University in St. Louis, Missouri (69)	0	0	0	0	0	1	0	1	0	0	0	0	0	0	0	0	0	0	0	0
New York Winthrop Hospital (91)	0	0	0	0	0	0	0	0	0	0	1	0	1	0	0	0	0	0	0	0
New York University, Langone Health (85)	0	0	0	0	0	0	0	0	0	0	11	0	11	0	0	0	0	0	0	0
San Francisco (89)	0	0	0	0	0	0	0	0	0	0	1	0	1	0	0	0	0	0	0	0
Livingstone, New Jersey (111)	0	0	0	0	0	0	0	0	0	0	2	1	1	0	0	0	0	0	0	0
Stanford University Hospital (115)	1	0	1	0	0	0	0	0	0	0	0	0	0	0	0	0	0	0	0	0
Weill Cornell Medicine, New York (118)	0	0	0	0	0	0	0	0	0	0	1	0	1	0	0	0	0	0	0	0
Michigan (123)	6	0	6	0	0	6	0	6	0	0	0	0	0	0	0	0	0	0	0	0
Maimonides Medical Center, Brooklyn (113)	0	0	0	0	0	0	0	0	0	0	68	0	48	20	0	0	0	0	0	0
**Honduras**																				
Hospital Escuela of Tegucigalpa (18)	0	0	0	0	0	0	0	0	0	0	1	0	1	0	0	0	0	0	0	0
**Sweden**																				
Southern General Hospital, Stockholm (20)	2	0	2	0	0	0	0	0	0	0	0	0	0	0	0	0	0	0	0	0
**Korea**																				
Daegu Fatimal Hospital (22)	0	0	0	0	0	1	0	1	0	0	0	0	0	0	0	0	0	0	0	0
**Turkey**																				
Ankara University (31)	0	0	0	0	0	0	0	0	0	0	0	0	0	0	0	1	0	1	0	0
**Italy**																				
IRCCS Fondazione Policlinico Universitario Agostino Gemelli (76)	1	1	0	0	0	0	0	0	0	0	0	0	0	0	0	1	0	1	0	0
Sant'Anna Hospital, Turin (79)	0	0	0	0	0	0	0	0	0	0	1	1	0	0	0	0	0	0	0	0
Palma (109)	4	0	3	1	0	0	0	0	0	0	0	0	0	0	0	0	0	0	0	0
Italian hospitals (117)	56	4	52	0	0	0	0	0	0	0	0	0	0	0	0	0	0	0	0	0
**Portugal**																				
Hospital Pedro Hispano in Porto (105)	0	0	0	0	0	0	0	0	0	0	10	0	10	0	0	0	0	0	0	0
**Australia**																				
Gold Coast University Hospital (45)	1	0	1	0	0	0	0	0	0	0	0	0	0	0	0	0	0	0	0	0
**Canada**																				
Mount Sinai Hospital (48A)	1	0	0	1	0	0	0	0	0	0	0	0	0	0	0	0	0	0	0	0
Toronto (103)	1	1	0	0	0	0	0	0	0	0	0	0	0	0	0	0	0	0	0	0
**France**																				
Antoine Béclère hospital (48B)	0	0	0	0	0	0	0	0	0	0	1	0	0	1	0	0	0	0	0	0
Spain																				
Madrid (125)		0	23	0	0															
**Lima, Peru**																				
British American Hospital (51)	0	0	0	0	0	1	1	0	0	0	0	0	0	0	0	0	0	0	0	0
**India**																				
Designated COVID hospital (58)	1	0	1	0	0	0	0	0	0	0	0	0	0	0	0	0	0	0	0	0
**Iran**																				
Tehran/Rasht/Qom/Zanjan (67)	0	0	0	0	0	6	1	5	0	2	2	0	0	0	0	0	0	0	0	0
Imam Khomeini Hospital, Sari (70)	0	0	0	0	0	1	1	0	0	0	0	0	0	0	0	0	0	0	0	0
Imam Reza Hospital of Tabriz (101)	0	0	0	0	0	0	0	0	0	0	1	0	1	0	0	0	0	0	0	0
**UK**																				
UKOSS (92)	0	0	0	0	0	0	0	0	0	0	265	12	253	0	2	0	0	0	0	0
**Belgium**																				
Cliniques Universitaires Saint Luc (100)	0	0	0	0	0	0	0	0	0	0	0	0	0	0	0	1	1	0	0	0
**Netherlands**																				
NethOSS (141)	33	0	33	0	0	16	0	16	0	0	0	0	0	0	0	0	0	0	0	0
**Total**	148	7	139	2	0	56	3	39	14	2	460	17	396	28	2	5	1	4	0	0

Study 43 excluded from table as no live neonates were included. In all, six babies from Study 67 in the table. Nine women and 11 babies included: six women underwent caesarean birth and delivered seven babies of whom 6/7 were born alive but 2/6 later died; of the six born alive, one tested positive for COVID‐19. One woman had a vaginal birth of a stillborn baby. Two women died before delivery. There were therefore four stillbirths and two neonatal deaths. For the purposes of this table, six babies are included.

**Table 4:** Isolation of neonate and COVID‐19 infection status in the neonate

Centre/hospital	Study number (Appendix S1)	Isolated from mother				Cared for in same room as mother				Not reported what approach was taken			
		Infected	Not infected	No test	Died	Infected	Not infected	No test	Died	Infected	Not infected	No test	Died
**China**													
Zhongnan Hospital of Wuhan University	1									0	9	0	0
Wuhan Children's Hospital	8									3	0	0	0
Maternal and Child Hospital of Hubei Province	30	0	3	14	0								
Central Hospital of Wuhan	73									0	23	0	0
Union Hospital, Tongji Medical College, Huazhong University of Science and Technology	2a and 6	0	3	0	0					0	3	11	0
Tongji Hospital, Tongji Medical College, Huazhong University of Science and Technology	15									1	2	4	0
First Affiliated Hospital of Sun Yat‐sen University	3									0	9	0	0
Renmin Hospital of Wuhan University	36 and 37	0	17	0	0					0	3	0	0
Affiliated Infectious Hospital of Soochow University, Suzhou	19	0	1	0	0								
Beijing YouAn Hospital	34									0	1	0	0
No. 2 People's Hospital of Hefei City Affiliated to Anhui Medical University	62	0	1	0	0								
**USA**													
New York Presbyterian Hospital Columbia	27					0	18	0	0				
MedStar Washington Hospital Center	21	0	1	0	0								
Good Samaritan Hospital, Cincinnati, Ohio	50									0	1	0	0
Hospital of the University of Pennsylvania	65									0	3	0	0
Washington University in St. Louis, Missouri	69									0	1	0	0
New York Winthrop Hospital	91									0	1	0	0
New York University, Langone Health	85									0	11	0	0
San Francisco	89									0	1	0	0
Livingstone, New Jersey	111	1	1	0	0								
Stanford University Hospital	115					0	1	0	0				
Weill Cornell Medicine, New York	118					0	1	0	0				
Michagan	123									0	12	0	0
Maimonides Medical Center, Brooklyn	113									0	48	20	0
**Honduras**													
Hospital Escuela of Tegucigalpa	18									0	1	0	0
**Sweden**													
Southern General Hospital, Stockholm	20					0	2	0	0				
**Korea**													
Daegu Fatimal Hospital	22	0	4	0	0								
**Turkey**													
Ankara University	31									0	1	0	0
**Italy**													
IRCCS Fondazione Policlinico Universitario Agostino Gemelli, Rome	76	1	1	0	0	0	0	0	0	0	0	0	0
Sant'Anna Hospital, Turin	79	1	0	0	0	0	0	0	0	0	0	0	0
Palma	109					0	3	1	0				
Italian hospitals	117					4	52	0	0				
**Portugal**													
Hospital Pedro Hispano in Porto (105)	105									0	10	0	0
**Australia**													
Gold Coast University Hospital	45					0	1	0	0				
**Canada**													
Mount Sinai Hospital	48a									0	0	1	0
Toronto (103)													
**France**													
Antoine Béclère Hospital	48B									0	0	1	0
**Spain**													
Madrid	125					0	23	0	0				
**Lima, Peru**													
British American Hospital	51	1	0	0	0								
**India**													
Designated COVID Hospital	58					0	1	0	0				
**Iran**													
Vali‐e‐asr Hospital, Zanjan	43	–	–	–	–	–	–	–	–	–	–	–	0
Tehran/Rasht/Qom/Zanjan	67	–	–	–	–	–	–	–	–	1	3	–	2
Imam Khomeini Hospital, Sari	70	1	0	0	0								
Imam Reza Hospital of Tabriz	101									0	1	0	0
**UK**													
UKOSS	92									12	253	0	2
**Belgium**													
Cliniques Universitaires Saint Luc	100	1	0	0	0								
**Netherlands**													
NethOSS	141									0	49	0	0
**Total**		6	32	14	0	4	102	1	0	17	446	37	2

Of the 666 neonates, 28 had confirmed COVID‐19 infection: full details are provided in Table S1. Due to a lack of virological testing at birth or in the first 12 hours of life, it was impossible to apply the classification proposed by Shah et al.^15^ Only eight had symptoms and of these, in four neonates the symptoms may have been related to prematurity.

In Table 2, data are shown on mode of delivery and neonate's infection status for 666 neonates as 11 women delivered twins. Of the 291 women who delivered vaginally, 8/292 (2.7%) neonates were found to be positive for COVID‐19. Of the 364 women who had a caesarean birth, 20/374 (5.3%) neonates were found to be positive for COVID‐19.

Of the 28 neonates with confirmed COVID‐19 infection, 7 were breastfed, 3 formula fed, 1 was given expressed breast milk and in 17 neonates the method of infant feeding was not reported. Overall, of the 666 neonates reviewed, 148 were breastfed, 56 formula fed, 5 given expressed breast milk and for 460 neonates the method of infant feeding was not reported.

Of the 28 neonates with confirmed COVID‐19 infection, 7 were kept isolated from their mother, 5 were cared for in the same room as their mother and for 16 neonates it was not reported what approach was taken. Overall, 52 neonates were kept isolated from their mother, 107 were cared for in the same room as their mother and for 502 neonates it was not reported what approach was taken.

## Discussion

### Main findings

We have shown that there has been a significant amount of duplicate reporting of cases of COVID‐19 from China. Second, neonatal COVID‐19 infection is uncommon, almost never symptomatic, and the rate of infection is no greater when the baby is born vaginally, breastfed or allowed contact with the mother. Very few infections have been reported in the newborns of COVID‐19‐positive mothers. Two were reported to have occurred despite isolation from the mother and in two it was not possible to tell what approach was taken for isolation. Some babies were born prematurely and eight infants were stillborn, two twins and two singletons died in the neonatal period but were COVID‐19‐negative.

To date, there have been 28 cases (Table S1) published where the possibility for vertical transmission to have occurred has been reported. To confirm definite vertical transmission, it has been proposed that detection of the virus by PCR in umbilical cord blood, neonatal blood collected within the first 12 hours of birth or amniotic fluid collected prior to rupture of membranes is needed.^15^ In no cases reported to date have these criteria been met, although some report negative testing. A few cases deserve special mention. Case 9 (Study 79) reports a positive nasopharyngeal swab in the neonate on the day of birth. The authors do not describe any procedure or care taken to clean the infant's oropharynx/mouth/nares/face prior to procuring the swab and we speculate that the presence of the virus may be due to contamination by maternal stool. Of note, the virus was not detected on repeat swab and the infant remained well. The presence of IgG would be maternal, and so again is not diagnostic. The UKOSS study reports 12/24 cases of possible vertical transmission. Limited information is given for the 12 neonates but 6/12 infants tested positive for COVID‐19 within 12 hours of birth. It is unclear what method of testing was used; if this was a nasopharyngeal swab without taking precautions to clean the infant prior to testing, the positive result may again be a result of contamination. In case 23 (Study 103) a positive nasopharyngeal swab in the neonate on the day of birth occurred after careful separation of the baby and cleansing of the baby prior to taking the swab. While the baby was PCR positive, and this is arguably the study that is closest to suggesting that vertical transmission is possible, there are still questions being raised about the results. Namely, in the supplemental data, only one of the gene targets was positive by PCR in the neonatal NP swab (rather than two or three), and the cycle threshold was high suggesting that there was minimal genetic material present. Some laboratories might actually call this an indeterminate result rather than positive, and therefore the result does not so clearly demonstrate vertical transmission.

Newborn infants can be infected in the first few hours of life but as very few are severely affected, it is likely that the benefits of contact with the mother and the ability to breastfeed outweigh the potential benefits of separation. For cases where the mother has suspected or confirmed COVID‐19 and the baby does not require care on the neonatal unit, guidelines including those in the UK and Canada advise skin‐to‐skin contact and breastfeeding if the mother uses hand hygiene precautions and (ideally) wears a surgical face mask.^5^, ^17^ The UNICEF guideline strongly recommends breastfeeding for all babies including preterm and sick babies. Our data support such recommendations. Maintaining physical separation of more than 2 m at other times is also recommended.^17^, ^18^

### Strengths and limitations

Despite having taken steps to remove duplicate reports, the present review is much larger than previous ones. The precision of our estimates is therefore greater. Reassuringly, our data after disambiguation for China agrees broadly with two recent multiple hospital reports from that country, one from Wuhan only^7^ and the other from a range of hospitals both inside and outside Wuhan.^19^

The studies analysed include a considerable number of case reports and hospital‐based series. Such reports have a high risk of being biased towards cases or findings of interest and it is important to reiterate that not all neonates born to COVID‐19‐positive women were tested for COVID‐19 infection. For example, studies may differentially report infected babies, or uninfected babies. However, we are reassured to find that our data are broadly in line with the two regional series reported so far (Lombardy^20^ and Netherlands [www.nvog.nl/actueel/registratie‐van‐covid‐19‐positieve‐zwangeren‐in‐nethoss/]).

Ideally data on rates of neonatal infection by type of care would come from registries, or population based studies. However, to date these have either not reported infection by mode of birth or feeding method,^7^ have found no infected babies (Nethoss), or have found few (three) infected babies.^20^

It is disappointing that the details of outcome and care of so many neonatal cases born to COVID‐19‐positive mothers have not been fully reported. This is a missed opportunity to confirm for neonatal and paediatric teams that babies are not likely to be vertically infected. It may be judged likely that babies would have been reported if there had been a poor outcome, but the general lack of rigour around taking samples at delivery or in the first few hours of life undermines this conclusion. Authors frequently failed to describe how the baby was looked after, often did not give details of testing, in particular not of the timing, and only occasionally were samples reported that were obtained at or shortly after birth. Timings described as ‘day 0’, ‘day 1’ and ‘24 hours’ also make it hard accurately to determine when samples were actually taken.

We report relatively few data from women with COVID‐19 infection acquired postnatally. It is plausible that neonates of such mothers may be at increased risk of infection, as they will not have received passive IgG transfer across the placenta.

While we have presented the data from a robust search of the literature for 655 women and 666 neonates, this still only includes 28 infected neonates and COVID‐19 is a new virus, so we caution the reader to interpret the data in light of this.

### Interpretation

The finding of low rates of neonatal infection after caesarean birth are in accord with the very first report of COVID‐19 in pregnancy.^21^ Other systematic reviews have been published on this topic^9^, ^10^, ^11^, ^12^, ^13^, ^14^, ^15^ and support our contention that vaginal delivery, breastfeeding and maternal–infant interaction are safe in the context of COVID‐19 disease.

## Conclusion

Our data suggest that COVID‐19 disease should not be an indication for caesarean birth, formula feeding or isolation of the infant from the mother. Caesareans should continue to be performed for the normal obstetric indications. Mothers who breastfeed and room‐in with their infants should continue to observe COVID‐19 hygiene precautions and wear a fluid‐resistant surgical face mask, if available, while feeding or caring for the baby. There is no evidence that isolating the baby from the mother is beneficial if such precautions are taken, and encouraging the baby to spend time with its mother is likely to help with breastfeeding and bonding. We recommend that separation only occurs where this is necessary for clinical indications.

Although further hospital‐based series and case reports will surely be published, better estimates of the risks of neonatal infection after different types of care are likely to come from registry studies which, as far as possible, include all cases in a geographical region or area. Such studies should indicate whether their cases are likely to overlap with other reports by listing the geographical and hospital sources of their cases. In an effort to provide confirmatory evidence on whether vertical transmission occurs in COVID‐19, sites seeing infants being born to mothers with COVID‐19 should take samples from the mother and baby shortly after birth, as described by Shah et al., and report these in the medical literature.

Neonatal COVID‐19 infection is uncommon, uncommonly symptomatic, and the rate of infection is no greater when the baby is born vaginally, breastfed or allowed contact with the mother.

### Disclosure of interests

None declared. Completed disclosure of interests forms are available to view online as supporting information.

### Contributions to authorship

JGT designed the study. KFW, JGT and KO conducted the literature search. KFW reviewed the literature and extracted the data. KFW, JGT and JD interpreted the data and wrote the manuscript. WL revised the manuscript to correct the process of disambiguation for Chinese hospitals. KO, JD and JLC reviewed the data. All authors reviewed and approved the manuscript.

### Details of ethics approval

No ethical approvals were required for this study of published work.

### Funding

No funding was received for this work.

### Acknowledgements

None.

## Supporting information


**Appendix S1.** Ripe‐tomato.org COVID‐19 in pregnancy academic publications. Curated list.
**Appendix S2.** Systematic review search strategy.
**Appendix S3.** Protocol for systematic review entitled ‘Clinical factors affecting maternal transmission of SARS‐COV‐2 to the neonate: a systematic review avoiding duplicate cases’.

**Figure S1.** Study flow chart.

**Table S1.** Virology results and summary of cases for babies with possible vertical transmission.
**Table S2.** Risk of bias assessment.

**Video S1.** Author Insights.

Supplementary Material

Supplementary Material

Supplementary Material

Supplementary Material

Supplementary Material

Supplementary Material

Supplementary Material
