# Supplementary material for: Maternal transmission of SARS‐COV‐2 to the neonate, and possible routes for such transmission: a systematic review and critical analysis
Source: BJOG. 2020 Jul 22;127(11):1324–36. doi: 10.1111/1471-0528.16362 (PMC7323034; doi:10.1111/1471-0528.16362)
Supplement: Supplementary file 1 — Appendix S1. Ripe‐tomato.org COVID‐19 in pregnancy academic publications. Curated list. Appendix S2. Systematic review search strategy. Appendix S3. Protocol for systematic review entitled ‘Clinical factors affecting maternal transmission of SARS‐COV‐2 to the neonate: a systematic review avoiding duplicate cases’. [file BJO-127-1324-s005.pdf]

Appendix S1. Ripe-tomato.org COVID-19 in pregnancy academic publications. Curated list.

1. Huijun Chen, Juanjuan Guo, Chen Wang, Fan Luo, Xuechen Yu, Wei Zhang, Jiafu Li, Dongchi Zhao, Dan Xu, Qing Gong, Jing Liao, Huixia Yang, Wei Hou, Yuanzhen Zhang. Clinical characteristics and intrauterine vertical transmission potential of COVID-19 infection in nine pregnant women: a retrospective review of medical records. Lancet. Volume 395, Issue 10226, 7–13 March 2020, Pages 809-815.

2.

a. Chen S, Huang B, Luo DJ, Li X, Yang F, Zhao Y, Nie X, Huang BX. Pregnant women with new coronavirus infection: a clinical characteristics and placental pathological analysis of three cases. Zhonghua Bing Li Xue Za Zhi. 2020 Mar 1; 49(0):E005. doi: 10.3760/cma.j.cn112151-20200225-00138.

b. Liu, W.; Wang, Q.; Zhang, Q.; Chen, L.; Chen, J.; Zhang, B.; Lu, Y.; Wang, S.; Xia, L.; Huang, L.; Wang, K.; Liang, L.; Zhang, Y.; Turtle, L.; Lissauer, D.; Lan, K.; Feng, L.; Yu, H.; Liu, Y.; Sun, Z. Coronavirus Disease 2019 (COVID-19) During Pregnancy: A Case Series. Preprints 2020, 2020020373

c. Yan Chen, Hua Peng, Lin Wang, Yin Zhao, Lingkong Zeng, Hui Gao and Yalan Liu. Infants Born to Mothers With a New Coronavirus (COVID-19) Front. Pediatr., 16 March 2020 | https://doi.org/10.3389/fped.2020.00104

3. Yangli Liu, Haihong Chen, Kejing Tang, Yubiao Guo. Clinical manifestations and outcome of SARS-CoV-2 infection during pregnancy J Infect. https://doi.org/10.1016/j.jinf.2020.02.0284.

4. Yang Li, Ruihong Zhao, Shufa Zheng, Xu Chen, Jinxi Wang, Xiaoli Sheng, Jianying Zhou, Hongliu Cai, Qiang Fang, Fei Yu, Jian Fan, Kaijin Xu, Yu Chen and Jifang Sheng Lack of Vertical Transmission of Severe Acute Respiratory Syndrome Coronavirus 2, China. Emerging Infectious Diseases. 2020 Apr [date cited]. https://doi.org/10.3201/eid2606.200287 DOI: 10.3201/eid2606.200287

5. Huaping Zhu, Lin Wang, Chengzhi Fang, Sicong Peng, Lianhong Zhang, Guiping Chang, Shiwen Xia, Wenhao Zhou. Clinical analysis of 10 neonates born to mothers with 2019-nCoV pneumonia. Translational Pediatrics Vol 9, No 1 (February 2020)

6. Dehan Liu, Lin Li, Xin Wu, Dandan Zheng, Jiazheng Wang, Lian Yang and Chuansheng Zheng. Pregnancy and Perinatal Outcomes of Women With Coronavirus Disease (COVID-19) Pneumonia: A Preliminary Analysis American Journal of Roentgenology: 1-6. 10.2214/AJR.20.23072

6a. Zhang Lu, Jiang Yan, Wei Min, et al. Analysis of pregnancy outcomes of pregnant women during the epidemic of new coronavirus pneumonia in Hubei [J / OL]. Chinese Journal of Obstetrics and Gynecology, 2020, 55 (2020-03-08). http://rs.yiigle.com/yufabiao/1184338.htm. DOI: 10.3760 / cma.j.cn112141-20200218-00111.

7. Rong Wen, Yue Sun, Quan-Sheng Xing A patient with SARS-CoV-2 infection during pregnancy in Qingdao, China Journal of Microbiology, Immunology and Infection Available online 10 March 2020 https://doi.org/10.1016/j.jmii.2020.03.004

8. Lingkong Zeng, Shiwen Xia, Wenhao Yuan, Kai Yan, Feifan Xiao, Jianbo Shao, Wenhao Zhou, Neonatal Early-Onset Infection With SARS-CoV-2 in 33 Neonates Born to Mothers With COVID-19 in Wuhan, China. JAMA Pediatr. Published online March 26, 2020. doi:10.1001/jamapediatrics.2020.0878

9. Kang X, Zhang R, He H, Yao Y, Zheng Y, Wen X, Zhu S. Anesthesia management in cesarean section for a patient with coronavirus disease 2019. Zhejiang Da Xue Bao Yi Xue Ban. 2020 May 25; 49(1):0. Chinese. PMID: 32207592

10. Cuifang Fan, Di Lei, Congcong Fang, Chunyan Li, Ming Wang, Yuling Liu, Yan Bao, Yanmei Sun, Jinfa Huang, Yuping Guo, Ying Yu, Suqing Wang Perinatal Transmission of COVID-19 Associated SARS-CoV-2: Should We Worry? Clinical Infectious Diseases, ciaa226, https://doi.org/10.1093/cid/ciaa226

11. Shaoshuai Wang, Lili Guo, Ling Chen, Weiyong Liu, Yong Cao, Jingyi Zhang, Ling Feng A Case Report of Neonatal 2019 Coronavirus Disease in China. Clinical Infectious Diseases, ciaa225, https://doi.org/10.1093/cid/ciaa225

12. Lan Dong, Jinhua Tian, Songming He, Chuchao Zhu, Jian Wang, Chen Liu, Jing Yang. Possible Vertical Transmission of SARS-CoV-2 From an Infected Mother to Her Newborn. JAMA. Published online March 26, 2020. doi:10.1001/jama.2020.4621

13. Hui Zeng, Chen Xu, Junli Fan, Yueting Tang, Qiaoling Deng, Wei Zhang, Xinghua Long. Antibodies in Infants Born to Mothers With COVID-19 Pneumonia. JAMA. Published online March 26, 2020. doi:10.1001/jama.2020.4861

14. Breslin N, Baptiste C, Miller R, Fuchs K, Goffman D, Gyamfi-Bannerman C, D'Alton M. COVID-19 in pregnancy: early lessons. Am J Obstet Gynecol. Available online 27 March 2020. https://doi.org/10.1016/j.ajogmf.2020.100111

15. Nan Yu, Wei Li, Qingling Kang, Zhi Xiong, Shaoshuai Wang, Xingguang Lin, Yanyan Liu, Juan Xiao, Haiyi Liu, Dongrui Deng, Suhua Chen, Wanjiang Zeng, Ling Feng, Jianli Wu. Clinical features and obstetric and neonatal outcomes of pregnant patients with COVID-19 in Wuhan, China: a retrospective, single-centre, descriptive study. Lancet Infect Dis 2020 Published Online March 24, 2020 https://doi.org/10.1016/S1473-3099 (20)30176-6

16. Yu Chen, Zhe Li, Yuan-Yuan Zhang, Wei-Hua Zhao, Zhi-Ying Yu. Maternal health care management during the outbreak of coronavirus disease 2019 (COVID-19) J Med Virol. 2020 Mar 26. doi: 10.1002/jmv.25787. [Epub ahead of print]

17. Chen S, Liao E, Shao Y. Clinical analysis of pregnant women with 2019 novel coronavirus pneumonia. J Med Virol. 2020 Mar 28. doi: 10.1002/jmv.25789. [Epub ahead of print]

18. Zambrano LI, Fuentes-Barahona IC, Bejarano-Torres DA, Bustillo C, Gonzales G, Vallecillo-Chinchilla G, Sanchez-Martínez FE, Valle-Reconco JA, Sierra M, Bonilla-Aldana DK, Cardona-Ospina JA, Rodríguez-Morales AJ. A pregnant woman with COVID-19 in Central America. Travel Medicine and Infectious Disease. Online 25 March 2020. https://doi.org/10.1016/j.tmaid.2020.101639

19. Xiaotong Wang, Zhiqiang Zhou, Jianping Zhang, Fengfeng Zhu, Yongyan Tang, Xinghua Shen. A case of 2019 Novel Coronavirus in a pregnant woman with preterm delivery. Clinical Infectious Diseases, ciaa200, https://doi.org/10.1093/cid/ciaa200

20. Gidlof S, Savchenko J, Brune, T, Josefsson H. COVID-19 in pregnancy with comorbidities: More liberal testing strategy is needed. Acta Obstetricia et Gynecologica Scandinavica

21. Iqbal SN Rachael Overcash R, Neggin Mokhtari N, Haleema Saeed H, Gold S, Auguste T, Mirza M-U, Ruiz M-E, Chahine JJ, Waga M, Wortmann G. An Uncomplicated Delivery in a Patient with Covid-19 in the United States New Engl J Med April 1, 2020 DOI: 10.1056/NEJMc2007605

22. Dong Hwan Lee, Jihyang Lee, Eunju Kim, Kyeongyoon Woo, Hak Youle Park, Jihyun An. Emergency cesarean section on severe acute respiratory syndrome coronavirus 2 (SARS- CoV-2) confirmed patient. Korean J Anesthesiol. https://doi.org/10.4097/kja.20116 [Epub ahead of print]

23. Mojgan Karimi-Zarchia, Hossein Neamatzadeh, Seyed Alireza Dastgheib, Hajar Abbasi, Seyed Reza Mirjalili, Thena Behforouz, Farzad Ferdosian, Reza Bahrami. Vertical Transmission of Coronavirus Disease 19 (COVID-19) from Infected Pregnant Mothers to Neonates: A Review. Fetal & Paed Pathol. https://doi.org/10.1080/15513815.2020.1747120

24. Lin Qiu, Xia Liu, Meng Xiao, Jing Xie, Wei Cao, Zhengyin Liu, Abraham Morse, Yuhua Xie, Taisheng Li, Lan Zhu. SARS-CoV-2 is not detectable in the vaginal fluid of women with severe COVID-19 infection. Clinical Infectious Diseases, ciaa375, https://doi.org/10.1093/cid/ciaa375

25. Report of the WHO-China Joint Mission on Coronavirus Disease 2019 (COVID-19) 16-24 February 2020. Report submitted 28 February 2020

26. Zeng Lingkong, Tao Xuwei, Yuan Wenhao, et al. The first neonatal coronavirus pneumonia in China [J]. Chinese Journal of Pediatrics, 2020,58 (04): 279-280. DOI: 10.3760 / cma.j.cn112140 -20200212-00081

27. Noelle Breslin, Caitlin Baptiste, Cynthia Gyamfi-Bannerman, Russell Miller, Rebecca Martinez, Kyra Bernstein, Laurence Ring, Ruth Landau, Stephanie Purisch , Alexander M. Friedman, Karin Fuchs, Desmond Sutton, Maria Andrikopoulou, Devon Rupley , Jean-Ju Sheen, Janice Aubey , Noelia Zork, Leslie Moroz, Mirella Mourad, Ronald Wapner, Lynn L. Simpson, Mary E. D'Alton, Dena Goffman. AJOG MFM.

28. Alexander Juusela, Munir Nazir Martin Gimovsky. Two Cases of COVID-19 Related Cardiomyopathy in Pregnancy. AJOG MFM Available online 3 April 2020, doi.org/10.1016/j.ajogmf.2020.100113

29. ICNARC COVID-19 report 2020-04-04.pdf https://www.icnarc.org/About/Latest-News/2020/04/04/Report-On-2249-Patients-Critically-Ill-With-Covid-19

30. Li N, Han L, Peng M, Lv Y, Ouyang Y, Liu K, Yue L, Li Q, Sun G, Chen L, Yang L. Maternal and neonatal outcomes of pregnant women with COVID-19 pneumonia: a case-control study. Clin Infect Dis. 2020 Mar 30. pii: ciaa352. doi: 10.1093/cid/ciaa352. [Epub ahead of print]

31. E. Kalafat E. Yaprak G. Cinar B. Varli S. Ozisik C. Uzun A. Azap A. Koc Lung ultrasound and computed tomographic findings in pregnant woman with COVID-19 Ultrasound Obstet Gynecol. First published: 06 April 2020 https://doi.org/10.1002/uog.22034

32. Ferrazzi EM, Frigerio L, Cetin I, Vergani P, Spinillo A, Prefumo F, Pellegrini E, Gargantini G.COVID-19 Obstetrics Task Force, Lombardy, Italy: executive management summary and short report of outcome. Int J Gynecol Obstet in press.

33. Preliminary Estimates of the Prevalence of Selected Underlying Health Conditions Among Patients with Coronavirus Disease 2019 — United States, February 12–March 28, 2020. MMWR Morb Mortal Wkly Rep 2020; 69:382–386. DOI: http://dx.doi.org/10.15585/mmwr.mm6913e2external icon.

34. Xiali Xiong, Hong Wei, Zhihong Zhang, Jing Chang, Xiaopeng Ma, Xiang Gao, Qiang Chen, Qiumei Pang. Vaginal Delivery Report of a Healthy Neonate Born to a Convalescent Mother with COVID-19 J Med Virol. 2020 Apr 10. doi: 10.1002/jmv.25857.

35. Zhi-Jiang Zhang, Xue-Jie Yu, Tao Fu, Yu Liu, Yan Jiang, Bing Xiang Yang, Yongyi Bi. Novel Coronavirus Infection in Newborn Babies Under 28 Days in China. European Respiratory Journal 2020; DOI: 10.1183/13993003.00697-2020

36. Rong Chen, Yuan Zhang, Lei Huang, Bi-heng Cheng, Zhong-yuan Xia, Qing-tao Meng, Safety and efficacy of different anesthetic regimens for parturients with COVID-19 undergoing Cesarean delivery: a case series of 17 patients. Can J Anesth https://doi.org/10.1007/s12630-020-01630-7

37. Suliman Khan, Liangyu Peng, Rabeea Siddique, Ghulam Nabi, Nawsherwan, Mengzhou Xue, Jianbo Liu, Guang Han. Infection Control & Hospital Epidemiology as part of the Cambridge Coronavirus Collection. March 2020. DOI: 10.1017/ice.2020.84

38. H. Liu, F. Liu and J. Li et al., Clinical and CT imaging features of the COVID-19 pneumonia: Focus on pregnant women and children, Journal of Infection, https://doi.org/10.1016/j.jinf.2020.03.007

39. Xiaoqing Wu, Ruihong Sun, Jianpu Chen, Yuanliang Xie, Shutong Zhang, Xiang Wang. Radiological findings and clinical characteristics of pregnant women with COVID-19 pneumonia In J Gynecol Obstet. In press doi: 10.1002/ijgo.13165

40. Khan S, Jun L, Nawsherwan Siddique R, Li Y, Han G, Xue M, Nabi G, Liu J, Association of COVID-19 infection with pregnancy outcomes in healthcare workers and general women, Clinical Microbiology and Infection, https://doi.org/10.1016/j.cmi.2020.03.034

41. Sutton, D, Fuchs, K, D'Alton M, Goffman D. Universal Screening for SARS-CoV-2 in Women Admitted for Delivery. New Engl J Med April 13, 2020. DOI: 10.1056/NEJMc2009316

42. The Nederlandse Vereniging voor Obstetrie en Gynaecologie (Dutch Association for Obstetrics and Gynecology) (NVOG). Registratie COVID-19 positieve zwangeren in NethOSS (Update registration COVID-19 positive pregnant women in NethOSS). Available https://www.nvog.nl/actueel/registratie-van-covid-19-positieve-zwangeren-in-nethoss/ Accessed 14 April.

43. Parisa Karami, Maliheh Naghavib, Abdolamir Feyzib, Mehdi Aghamohammadic, Mohammad Sadegh Novinc, Ahmadreza Mobaiend, Mohamad Qorbanisania, Aida Karamia, Amir Hossein Norooznezhad. Travel Medicine and Infectious Disease, in press. https://doi.org/10.1016/j.tmaid.2020.101665

44. Hui yang, Guoqiang Sun, Fei Tang, Min Peng, Ying Gao, Jing Peng, Hui Xie, Yun Zhao, Zhichun Jin, Clinical Features and Outcomes of Pregnant Women Suspected of Coronavirus Disease 2019, Journal of Infection (2020), doi: https://doi.org/10.1016/j.jinf.2020.04.003

45. Lowe B, Bopp B. COVID-19 vaginal delivery – a case report. ANZOG 15 April 2020. https://doi.org/10.1111/ajo.13173

46. 16 April update of NethOSS available here. https://www.nvog.nl/actueel/registratie-van-covid-19-positieve-zwangeren-in-nethoss/ Accessed 20 April 2020

47. COVID-19: epidemiological update of April 16, 2020 Santé Publique France. Available here. https://ripetomato2uk.files.wordpress.com/2020/03/covid19_pe_20200418.pdf Accesed 20 April 2020

48. Evangelia Vlachodimitropoulou Koumoutsea, Alexandre J. Vivanti, Nadine Shehata, Alexandra Benachi, Agnes Le Gouez, Celine Desconclois, Wendy Whittle, John Snelgrove, Kinga Ann Malinowski. COVID19 and acute coagulopathy in pregnancy. J Thrombosis Haemostasis. 17 April 2020. https://doi.org/10.1111/jth.14856

49. Tekbali A, Grünebaum A, Saraya A, McCullough L, Bornstein E, Chervenak FA. Pregnant versus non-pregnant SARS-CoV-2 and COVID-19 Hospital Admissions: The first 4 weeks in New York, American Journal of Obstetrics and Gynecology (2020), doi: https://doi.org/10.1016/j.ajog.2020.04.012.

50. Schnettler WT, Al Ahwel Y, Suhag A, Severe ARDS in COVID-19-infected pregnancy: obstetric and intensive care considerations American Journal of Obstetrics & Gynecology MFM (2020), doi: https://doi.org/10.1016/j.ajogmf.2020.100120.

51. Maria Claudia Alzamora, Tania Paredes, David Caceres, Camille M. Webb, Luis M. Valdez, Mauricio La Rosa. Severe COVID-19 during Pregnancy and Possible Vertical Transmission. Am J Perinatal. Online April 18. DOI https://doi.org/10.1055/s-0040-1710050.

52. ICNARC update 17 April

53. González Romero D, Ocampo Pérez J, González Bautista L, Santana-Cabrera L. Pronóstico perinatal y de la paciente embarazada con infección por COVID-19. Revista Clínica Española Available online 17 April 2020, https://doi.org/10.1016/j.rce.2020.04.006

54. Wu, C., Yang, W., Wu, X. et al. Clinical Manifestation and Laboratory Characteristics of SARS-CoV-2 Infection in Pregnant Women. Virol. Sin. (2020). https://doi.org/10.1007/s12250-020-00227-0

55. Lian Chen, Qin Li, Danni Zheng, Hai Jiang, Yuan Wei, Li Zou, Ling Feng, Guoping Xiong, Guoqiang Sun, Haibo Wang, Yangyu Zhao, Jie Qiao, Clinical Characteristics of Pregnant Women with Covid-19 in Wuhan, China. New Engl J Med. April 17, 2020 DOI: 10.1056/NEJMc2009226

56. Pu Yang, Xia Wang, Pin Liu, Cong Wei, Bingyan He, Junwen Zheng, Dongchi Zhao. Clinical characteristics and risk assessment of newborns born to mothers with COVID-19 Journal of Clinical Virology Volume 127, June 2020, 104356. https://doi.org/10.1016/j.jcv.2020.104356

57. Yan J, Guo J, Fan C, Juan J, Yu X, Li J, Feng L, Li C, Chen H, Qiao Y, Lei D, Wang C, Xiong G, Xiao F, He W, Pang Q, Hu X, Wang S, Chen D, Zhang Y, Poon LC, Yang H, Coronavirus disease 2019 (COVID-19) in pregnant women: A report based on 116 cases, American Journal of Obstetrics and Gynecology (2020), doi: https://doi.org/10.1016/j.ajog.2020.04.014.

58. K. Aparna Sharma, Rajesh Kumari, Garima Kachhawa, Anjolie Chhabra, Ramesh Agarwal, Akash Sharma, Neerja Bhatla. Management of the first patient with confirmed COVID-19 in pregnancy in India: From guidelines to frontlines. Int J Gynecol Obstet. In press. doi:10.1002/ijgo.13179

59. Nan Yu, Wei Li, Qingling Kang, Wanjiang Zeng, Ling Feng, Jianli Wu. No SARS-CoV-2 detected in amniotic fluid in mid-pregnancy. Lancet Infect Dis 2020 Published Online April 22, 2020 https://doi.org/10.1016/S1473-3099 (20)30320-0

60. Paul C. Browne, Jennifer B. Linfert, Emilio Perez-Jorge. Successful Treatment of Preterm Labor in Association with Acute COVID-19 Infection. Am J Perinatol. Published Online 24 April 2020. DOI: 10.1055/s-0040-1709993

61. William S VINTZILEOS, Jolene MUSCAT, Eva HOFFMANN, Duc VO, Nicole S JOHN, Rosanne VERTICHIO, Anthony M VINTZILEOS, Screening all pregnant women admitted to Labor and Delivery for the virus responsible for 2 COVID-19 American Journal of Obstetrics & Gynecology. In press.

62. Lu D, Sang L, Du S, Li T, Chang Y, Yang XA. Asymptomatic COVID-19 infection in late pregnancy indicated no vertical transmission. J Med Virol. 2020 Apr 24. doi: 10.1002/jmv.25927. [Epub ahead of print]

63. ICNARC 24 April update

64. Enrico Ferrazzi, Luigi Frigerio, Valeria Savasi, Patrizia Vergani, Federico Prefumo, Santa Barresi, Stefano Bianchi, Elena Ciriello, Fabio Facchinetti, Maria Teresa Gervasi, Enrico Iurlaro, Alessandra Kustermann, Giovanna Mangili, Fabio Mosca, Luisa Patanè, Donata Spazzini, Arsenio Spinillo, Giuseppe Trojano, Michele Vignali, Antonella Villa, GianVincenzo Zuccott,i Fabio Parazzini, Irene Cetin. Vaginal delivery in SARS-CoV-2 infected pregnant women in Northern Italy: a retrospective analysis. BJOG 27 April 2020 https://doi.org/10.1111/1471-0528.16278

65. Adi HIRSHBERG,, Adina R. KERN-GOLDBERGER, Lisa D. LEVINE,, Rebecca PIERCE-WILLIAMS, William R. SHORT, Samuel PARRY, Vincenzo BERGHELLA, Jourdan E. TRIEBWASSER, Sindhu K. SRINIVAS. Care of critically ill pregnant patients with COVID-19: a case series. Am J Obstet Gynecol. In press.

66. Amorim MMR, Takemoto MLS, Fonseca EB. Maternal Deaths with Covid19: a different outcome from mid to low resource countries? Am J Obstet Gynnecol. In press.

67. Sedigheh Hantoushzadeh, Alireza A. Shamshirsaz, Ashraf Aleyasin, Maxim D Seferovic, Soudabeh Kazemi Aski, Sara E. Arian, Parichehr Pooransari, Fahimeh Ghotbizadeh, Soroush Aalipour, ,Zahra Soleimani, Mahsa Naemi, Behnaz Molaei, Roghaye Ahangari, Mohammadreza Salehi, Atousa Dabiri Oskoei, Parisa Pirozan, Roya Faraji Darkhaneh, Mahboobeh Gharib Laki, Ali Karimi Farani, Shahla Atrak, Mir Mohammad Miri, Mehran Kouchek, Seyedpouzhia Shojaei, Fahimeh Hadavand, Fatemeh Keikha, Maryam Sadat Hosseini, Sedigheh Borna, Shideh Ariana, Mamak Shariat, Alireza Fatemi, Behnaz Nouri, Seyed Mojtaba Nekooghadam, Kjersti Aagaard. Maternal Death Due to COVID-19 Disease. Am J Obstet Gynecol. In press. https://doi.org/10.1016/j.ajog.2020.04.030

68. Annemarie B Docherty, Ewen M Harrison, Christopher A Green, Hayley E Hardwick, Riinu Pius, Lisa Norman, Karl A Holden, Jonathan M Read, Frank Dondelinger, Gail Carson, Laura Merson, James Lee, Daniel Plotkin, Louise Sigfrid, Sophie Halpin, Clare Jackson, Carrol Gamble, Peter W Horby, Jonathan S Nguyen-Van-Tam, Jake Dunning, Peter JM Openshaw, J Kenneth Baillie, Malcolm Gracie Semple. Features of 16,749 hospitalised UK patients with COVID-19 using the ISARIC WHO Clinical Characterisation Protocol. medRxiv 2020.04.23.20076042; doi: https://doi.org/10.1101/2020.04.23.20076042

69. Jeannie C Kelly, Michael Dombrowksi, Micaela O'neil-Callahan, Annessa S Kernberg, Antonina I Frolova, Molly J Stout. False-Negative COVID-19 Testing: Considerations in Obstetrical Care. American Journal of Obstetrics & Gynecology MFM Available online 28 April 2020, https://doi.org/10.1016/j.ajogmf.2020.100130

70. Marzieh Zamaniyan, Aghdas Ebadi, Samaneh Aghajanpoor Mir, Zahra Rahmani, Mohammadreza Haghshenas, Setareh Azizi. Preterm delivery in pregnant woman with critical COVID-19 pneumonia and vertical transmission. Prenatal diagnosis. First published: 17 April 2020 https://doi.org/10.1002/pd.5713

71. Lin Li, Dehan Liu, Lian Yang. Follow-Up Information About the Four Pregnant Patients With Coronavirus Disease (COVID-19) Pneumonia Who Were Still in the Hospital at the End of Our Study American Journal of Roentgenology: W1-W2. 10.2214/AJR.20.23247

72. David Baud, Gilbert Greub, Guillaume Favre, Carole Gengler, Katia Jaton, Estelle Dubruc, Léo Pomar. Second-Trimester Miscarriage in a Pregnant Woman With SARS-CoV-2 Infection. JAMA. Published online April 30, 2020. doi:10.1001/jama.2020.7233

73. Qiancheng X, Jian S, Lingling P, Lei H, Xiaogan J, Weihua L, Gang Y, Shirong L, Zhen W, GuoPing X, Lei Z, The sixth batch of Anhui medical team aiding Wuhan for COVID-19, Coronavirus disease 2019 in pregnancy, International Journal of Infectious Diseases (2020), doi: https://doi.org/10.1016/j.ijid.2020.04.065

74. Joana LYRA, Rita VALENTE, Marta ROSÁRIO, Mariana GUIMARÃES. Acta Med Port 2020 xxx; 33(AOP): xxx-xxx ▪ https://doi.org/10.20344/amp.13883

75. Jing Liao, Xiaoyan He, Qing Gong, Lingyun Yang, Chunhua Zhou, Jiafu Li. Analysis of vaginal delivery outcomes among pregnant women in Wuhan, China during the COVID-19 pandemic. First published: 29 April 2020 https://doi.org/10.1002/ijgo.13188

76. Danilo Buonsenso, Simonetta Costa, Maurizio Sanguinetti, Paola Cattani, Brunella Posteraro, Simona Marchetti, Brigida Carducci, Antonio Lanzone, Enrica Tamburrini, Giovanni Vento, Piero Valentini. Neonatal Late Onset Infection with Severe Acute Respiratory Syndrome Coronavirus 2. Am J Perinatol https://doi.org/10.1055/s-0040-1710541.

77. ICNARC 1st May update

78. Inchingolo R, Smargiassi A, Moro F, Buonsenso D, Salvi S, Del Giacomo P, Scoppettuolo G, Demi L, Soldati G, Testa AC, The Diagnosis of Pneumonia in a Pregnant Woman with COVID-19 Using Maternal Lung Ultrasound. American Journal of Obstetrics and Gynecology (2020), doi: https://doi.org/10.1016/j.ajog.2020.04.020.

79. A. Carosso, et al., Pre-labor anorectal swab for SARS-CoV-2 in COVID-19 pregnant patients: is it time to think about it? Eur J Obstet Gynecol (2020), https://doi.org/10.1016/j.ejogrb.2020.04.023

80. Wu Y, Liu C, Dong L, Zhang C, Chen Y, Liu J, Zhang C, Duan C, Zhang H, Mol BW, Dennis CL, Yin T, Yang J, Huang H. Coronavirus disease 2019 among pregnant Chinese women: Case series data on the safety of vaginal birth and breastfeeding. BJOG. 2020 May 5. doi: 10.1111/1471-0528.16276. [Epub ahead of print]

81. Li J, Wang Y, Zeng Y, Song T, Pan X, Jia M, He F, Hou L, Li B, He S, Chen D. Critically ill pregnant patient with COVID-19 and neonatal death within two hours of birth. Int J Gynaecol Obstet. 2020 May 5. doi: 10.1002/ijgo.13189.

82. Asma KHALIL, Robert HILL, Shamez LADHANI, Katherine PATTISSON, Pat O'BRIEN. SARS-CoV-2 in pregnancy: symptomatic pregnant women are only the tip of the iceberg. Am J Obstet Gynecol. in press.

83. Matthew J. Blitz, Amos Grünebaum, Asma Tekbali, Eran Bornstein, Burton Rochelson, Michael Nimaroff, Frank A. Chervenak. Intensive Care Unit Admissions for Pregnant and Non-Pregnant Women with COVID-19. Am J Obstet Gynecol in press.

84. Katy Kuhrt, Jess McMicking, Surabhi Nanda, Catherine Nelson Piercy, Andrew Shennan. Placental abruption in a twin pregnancy at 32 weeks' gestation complicated by COVID-19, without vertical transmission to the babies. Am J Obstet Gynecol in press.

85. Christina A. Penfield, Sara G. Brubaker, Meghana A. Limaye, Jennifer Lighter, Adam J. Ratner Kristen M. Thomas, Jessica Meyer, Ashley S. Roman. Detection of SARS-COV-2 in Placental and Fetal Membrane Samples. Am J Obstet Gynecol. In press.

86. Rebecca A.M. Pierce-Williams, Julia Burd, Laura Felder, Rasha Khoury, Peter S. Bernstein, Karina Avila, Christina A. Penfield, Ashley S. Roman, Chelsea A. DeBolt, Joanne L. Stone, Angela Bianco, Adina R. Kern-Goldberger, Adi Hirshberg, Sindhu K. Srinivas,, Jenani S. Jayakumaran, Justin S. Brandt, Hannah Anastasio, Meredith Birsner, Devon S. O'Brien, Harish M. Sedev, Cara D. Dolin, William T. Schnettler, Anju Suhag, Shabani Ahluwalia, Reshama S. Navathe, Adeeb Khalifeh, Kathryn Anderson, Vincenzo Berghella. Clinical course of severe and critical COVID-19 in hospitalized pregnancies: a US cohort study. Am J Obstet Gynecol. In press.

87. L. Hong, N. Smith, M. Keerthy, et al., Severe COVID-19 infection in pregnancy requiring intubation without preterm delivery: A case report. Case Reports in Women's Health (2020), https://doi.org/10.1016/j.crwh.2020.e00217

88. Collin J, Byström E, Carnahan A, Ahrne M. Pregnant and postpartum women with SARS-CoV-2 infection in intensive care in Sweden. Acta Obstet Gynecol Scand. 2020 May 9. doi: 10.1111/aogs.13901. [Epub ahead of print]

89. Blauvelt CA, Chiu C, Donovan AL, Prahl M, Shimotake TK, George RB, Schwartz BS, Farooqi NA, Ali SS, Cassidy A, Gonzalez JM, Gaw SL. Acute Respiratory Distress Syndrome in a Preterm Pregnant Patient With Coronavirus Disease 2019 (COVID-19). Obstet Gynecol. 2020 May 8. doi: 10.1097/AOG.0000000000003949. [Epub ahead of print]

90. The 8th May update (click here or ICNARC COVID-19 report 2020-05-08.pdf) contains 21 currently, and 30 recently, pregnant women critically ill with Covid-19. Of these 9 and 16 respectively had received advanced respiratory support. Excluded from running totals.

91. Gabriela N. ALGARROBA,, Patricia REKAWEK, Sevan A. VAHANIAN, Poonam KHULLAR, Thomas PALAIA, Morgan R. PELTIER, Martin R. CHAVEZ, Anthony M. VINTZILEOS. Visualization of SARS-CoV-2 virus invading the human placenta using electron microscopy. Am J Obstet Gynecol. in press.

92. Marian Knight, Kathryn Bunch, Nicola Vousden, Eddie Morris, Nigel Simpson, Chris Gale, Patrick O'Brien, Maria Quigley, Peter Brocklehurst, Jennifer J Kurinczuk. Characteristics and outcomes of pregnant women hospitalised with confirmed SARS-CoV-2 infection in the UK: a national cohort study using the UK Obstetric Surveillance System (UKOSS May 11) in press.

93. A. Govind, et al., Re: Novel Coronavirus COVID-19 in late pregnancy: Outcomes of first nine cases in an inner city London hospital, Eur J Obstet Gynecol (2020), https://doi.org/10.1016/j.ejogrb.2020.05.004

94. Polonia-Valente R, Moucho M, Tavares M, Vilan A, Montenegro N, Rodrigues T. Vaginal delivery in a woman infected with SARS-CoV-2 – the first case reported in Portugal. European Journal of Obstetrics & Gynecology & Reproductive Biology (2020), doi: https://doi.org/10.1016/j.ejogrb.2020.05.007

95. Justin Mulveya, Cynthia M.Magro, Lucy X Ma, Gerard J Nuovo, Rebecca N Baergen. A mechanistic analysis placental intravascular thrombus formation in COVID-19 patients. Annals of Diagnostic Pathology Volume 46, June 2020, https://doi.org/10.1016/j.anndiagpath.2020.151529

96. J. Justin Mulveya, Cynthia M.Magro, Lucy X Ma, Gerard J Nuovo, Rebecca N Baergen. Analysis of complement deposition and viral RNA in placentas of COVID-19 patients. Annals of Diagnostic Pathology Volume 46, June 2020, https://doi.org/10.1016/j.anndiagpath.2020.151530

97. Vibert F, Kretz M, Thuet V, Barthel F, De Marcillac F, Deruelle P, Lecointre L, Prone positioning and high-flow oxygen improved respiratory function in a 25-week pregnant woman with COVID-19. European Journal of Obstetrics & Gynecology and Reproductive Biology (2020). doi: https://doi.org/10.1016/j.ejogrb.2020.05.022

98. Melissa H Rosen, MD, Jordan Axelrad, MD, MPH, David Hudesman, MD, David T Rubin, MD, Shannon Chang, MD, Management of Acute Severe Ulcerative Colitis in a Pregnant Woman With COVID-19 Infection: A Case Report and Review of the Literature, Inflammatory Bowel Diseases, , izaa109, https://doi.org/10.1093/ibd/izaa109

99. Baergen, R. N., & Heller, D. S. (2020). Placental Pathology in Covid-19 Positive Mothers: Preliminary Findings. Pediatric and Developmental Pathology, 23(3), 177–180. https://doi.org/10.1177/1093526620925569

100. Fiammetta Piersigilli, Katherine Carkeek, Catheline Hocq, Bénédicte van Grambezen, Corinne Hubinont, Olga Chatzis, Dimitri Van der Linden, Olivier Danhaive COVID-19 in a 26-week preterm neonate. Lancet Child Adolesc Health 2020 Published Online May 7, 2020 https://doi.org/10.1016/S2352-4642 (20)30140-1

101. Taghizadieh A, Mikaeili H, Ahmadi M, Valizadeh H, Acute kidney injury in pregnant women following SARS-CoV-2 infection: A case report from Iran, Respiratory Medicine Case Reports (2020), doi: https://doi.org/10.1016/j.rmcr.2020.101090.

102. Erica M. LOKKEN et al. Clinical Characteristics of 46 Pregnant Women with a SARS-CoV-2 Infection in Washington State. Am J Obstet Gynecol. 2020 May 19 doi: 10.1016/j.ajog.2020.05.031 [Epub ahead of print] PMCID: PMC7234933 PMID: 32439389

103. Citation: Maksim Kirtsman, Yenge Diambomba, Susan M. Poutanen, Ann K. Malinowski, Evangelia Vlachodimitropoulou, W. Tony Parks, Laura Erdman, Shaun K. Morris, Prakesh S. Shah. Probable congenital SARS-CoV-2 infection in a neonate born to a woman with active SARS-CoV-2 infection CMAJ 2020. doi: 10.1503/cmaj.200821; early-released May 14, 2020

104. ICNARC 15th May update

105. D´oria M, Peixinho C, Laranjo M, Varejao AM, Silva PT. Covid-19 during pregnancy: a case series from an universally tested population from the north of Portugal. Eur J Obstet Gynecol & Reprod Biol (2020). doi: https://doi.org/10.1016/j.ejogrb.2020.05.029

106. Cooke WR, Billett A, Gleeson S, Jacques A, Place K, Siddall J, Walden A, Soulsby K. SARS-CoV-2 infection in very preterm pregnancy: experiences from two cases, Eur J Obstet Gynecol & Reprod Biol (2020), doi: https://doi.org/10.1016/j.ejogrb.2020.05.025

107. Ahmed, I., Azhar, A., Eltaweel, N. and Tan, B.K. (2020), First Covid-19 maternal mortality in the UK associated with thrombotic complications. Br J Haematol. Accepted Author Manuscript. doi:10.1111/bjh.16849

108. Hui Yang, Bin Hu, Sudong Zhan, Li-ye Yang, Guoping Xiong. Effects of SARS-CoV-2 infection on pregnant women and their infants: A retrospective study in Wuhan, China. Arch Pathol Lab Med. In press. doi: 10.5858/arpa.2020-0232-SA

109. Serafina Perrone, Michela Deolmi, Maurizio Giordano, Tiziana D'Alvano, Lucia Gambini, Mara Corradi, Tiziana Frusca, Tullio Ghi, Susanna Esposito. Report of a series of healthy term newborns from convalescent mothers with COVID-19. Acta Biomed 2020; Vol. 91, N. 2: 251-255 DOI: 10.23750/abm.v91i2.9743

110. Panichaya P, Thaweerat W, Uthaisan J. Prolonged viral persistence in COVID-19 second trimester pregnant patient, Eur J Obstet & Gynecology & Reprod Biol (2020), doi: https://doi.org/10.1016/j.ejogrb.2020.05.030

111. H. Mehta, S. Ivanovic, A. Cronin, et al., Novel coronavirus related acute respiratory distress syndrome in a patient with twin pregnancy: A case report. Case Rep Women's Health (2020), https://doi.org/10.1016/j.crwh.2020.e00220

112. J. Anderson, J. Schauer, S. Bryant, et al., The use of convalescent plasma therapy and remdesivir in the successful management of a critically ill obstetric patient with novel coronavirus 2019 infection: A case report. Case Rep Women's Health (2020), https://doi.org/10.1016/j.crwh.2020.e00221

113. Viktoriya London, Rodney McLaren Jr., Fouad Atallah, Catherine Cepeda, Sandra McCalla, Nelli Fisher, Janet L. Stein, Shoshana Haberman, Howard Minkoff. The Relationship between Status at Presentation and Outcomes among Pregnant Women with COVID-19. Am J Perinatol. DOI https://doi.org/10.1055/s-0040-1712164. ISSN 0735-1631. Online April 27, 2020

114. Patanè L, Morotti D, Giunta MR, Sigismondi C, Piccoli MG, Frigerio L, Mangili G, Arosio M, Cornolti G, Vertical transmission of COVID-19: SARS-CoV-2 RNA on the fetal side of the placenta in pregnancies with COVID-19 positive mothers and neonates at birth, American Journal of Obstetrics & Gynecology MFM (2020), doi: https://doi.org/10.1016/j.ajogmf.2020.100145.

115. Joudi N, Henkel A, Lock WS, Lyell D, Preeclampsia Treatment in SARSCoV-2, American Journal of Obstetrics & Gynecology MFM (2020), doi: https://doi.org/10.1016/j.ajogmf.2020.100146.

116. Miller, Emily S. MD, MPH; Grobman, William A. MD, MBA; Sakowicz, Allie MS; Rosati, Jessica MSN, RN; Peaceman, Alan M. MD Clinical Implications of Universal Severe Acute Respiratory Syndrome Coronavirus 2 (SARS-CoV-2) Testing in Pregnancy, Obstetrics & Gynecology: May 19, 2020 - Volume Publish Ahead of Print - Issue - doi: 10.1097/AOG.0000000000003983

117. Savasi, Valeria M. MD, PhD; Parisi, Francesca MD, PhD; Patanè, Luisa MD; Ferrazzi, Enrico MD; Frigerio, Luigi MD, PhD; Pellegrino, Antonio MD; Spinillo, Arsenio MD; Tateo, Saverio MD; Ottoboni, Mariacristina MD; Veronese, Paola MD, PhD; Petraglia, Felice MD; Vergani, Patrizia MD; Facchinetti, Fabio MD; Spazzini, Donata MD; Cetin, Irene MD, PhD Clinical Findings and Disease Severity in Hospitalized Pregnant Women With Coronavirus Disease 2019 (COVID-19), Obstetrics & Gynecology: May 19, 2020 - Volume Publish Ahead of Print - Issue - doi: 10.1097/AOG.0000000000003979

118. Victoria Vallejo, John G. Ilagan. A Postpartum Death Due to Coronavirus Disease 2019 (COVID-19) in the United States. Obstet Gynecol 2020;00:1–4 DOI: 10.1097/AOG.0000000000003950

119. Bianco, Angela; Buckley, Ayisha B; Overbey, Jessica; Smilen, Scott; Wagner, Brian; Dinglas, Cheryl; Loudon, Holly; Garely, Alan; Brodman, Michael; Stone, Joanne. Testing of Patients and Support Persons for Coronavirus Disease 2019 (COVID-19) Infection Before Scheduled Deliveries, Obstetrics & Gynecology: May 19, 2020 – Volume Publish Ahead of Print – Issue – doi: 10.1097/AOG.0000000000003985

120. 14 May update of NethOSS

121. Mariam Naqvi, Richard M Burwick, John A Ozimek, Naomi H Greene, Sarah J Kilpatrick, Melissa S Wong. Severe Acute Respiratory Syndrome Coronavirus 2 (SARS-CoV-2) Universal Testing Experience on a Los Angeles Labor and Delivery Unit, Obstetrics & Gynecology: May 19, 2020 - Volume Publish Ahead of Print - Issue - doi: 10.1097/AOG.0000000000003987

122. Burton Rochelson, Michael Nimaroff, Adriann Combs, Benjamin Schwartz, Natalie Meirowitz, Nidhi Vohra, Victor R. Klein, Orlando Santandreu, Mitchell Kramer, Navid Mootabar, Eli Serur, Lisa Spiryda, Scott Berlin. Frank Chervenak. The Care of Pregnant Women During the COVID-19 Pandemic – Response of a Large Health System in Metropolitan New York. J Perinat Med. 2020 May 20;/j/jpme.ahead-of-print/jpm-2020-0175/jpm-2020-0175.xml. doi: 10.1515/jpm-2020-0175. Online ahead of print.

123. Farnoosh Qadri & Federico Mariona (2020) Pregnancy affected by SARS-CoV-2 infection: a flash report from Michigan. J Mat-Fetal & Neonatal Med. DOI: 10.1080/14767058.2020.1765334

124. Elisheva D Shanes, Leena B Mithal, Sebastian Otero, Hooman A Azad, Emily S Miller, Jeffery A Goldstein. Placental Pathology in COVID-19, Am J Clin Pathol. aqaa089, https://doi.org/10.1093/ajcp/aqaa089 Published: 22 May 2020

125. Pereira, A., Cruz-Melguizo, S., Adrien, M., Fuentes, L., Marin, E. and Perez-Medina, T. (2020), Clinical course of Coronavirus Disease-2019 (COVID-19) in pregnancy. Acta Obstet Gynecol Scand. Accepted Author Manuscript. doi:10.1111/aogs.13921

126. Docherty Annemarie B, Harrison Ewen M, Green Christopher A, Hardwick Hayley E, Pius Riinu, Norman Lisa et al. Features of 20 133 UK patients in hospital with covid-19 using the ISARIC WHO Clinical Characterisation Protocol: prospective observational cohort study. BMJ 2020; 369 :m1985

127. Rüdiger Groß, Carina Conzelmann, Janis A Müller, Steffen Stenger, Karin Steinhart, Frank Kirchhoff, Jan Münch. Detection of SARS-CoV-2 in human breastmilk. Lancet. Published online May 21, 2020 https://doi.org/10.1016/S0140-6736(20)31181-8

128. Ceulemans, D., Thijs, I., Schreurs, A., Vercammen, J., Lannoo, L., Deprest, J., Richter, J., De Catte, L. and Devlieger, R. (2020), Screening for COVID -19 at childbirth: does it deliver?. Ultrasound Obstet Gynecol. Accepted Author Manuscript. doi:10.1002/uog.22099

129. Duffy, Cassandra R. MD, MPH; Hart, Jessica M. MD; Modest, Anna M. PhD; Hacker, Michele R. ScD; Golen, Toni MD; Li, Yunping MD, MSc; Zera, Chloe MD, MPH; Shainker, Scott A. DO, MS; Mehrotra, Preeti MD, MPH; Zash, Rebecca MD; Wylie, Blair J. MD, MPH Lymphopenia and Severe Acute Respiratory Syndrome Coronavirus 2 (SARS-CoV-2) Infection Among Hospitalized Obstetric Patients, Obstetrics & Gynecology: May 19, 2020 – Volume Publish Ahead of Print – Issue – doi: 10.1097/AOG.0000000000003984

130. Man Wai Tang, Erfan Nur, Bart J. Biemond. Immune Thrombocytopenia during Pregnancy due to COVID-19. Am J Haematol. First published:23 May 2020 https://doi.org/10.1002/ajh.25877

131. Alisa Fox, Jessica Marino, Fatima Amanat, Florian Krammer, Jennifer Hahn-Holbrook, Susan Zolla-Pazner, Rebecca L Powell. Evidence of a significant secretory-IgA-dominant SARS-CoV-2 immune response in human milk following recovery from COVID-19 doi: https://doi.org/10.1101/2020.05.04.20089995

132. Katherine H. Campbell, Jean M. Tornatore, Kirsten E. Lawrence, Jessica L. Illuzzi, L. Scott Sussman, Heather S. Lipkind, Christian M. Pettker. Prevalence of SARS-CoV-2 Among Patients Admitted for Childbirth in Southern Connecticut. JAMA. Published online May 26, 2020. doi:10.1001/jama.2020.8904

133. Gagliardi L, Danieli R, Suriano G, Vaccaro A, Tripodi G, Rusconi F, Ramenghi LA, Universal SARS-CoV-2 testing of pregnant women admitted for delivery in two Italian regions. Am J Obstet Gynecol (2020), doi: https://doi.org/10.1016/j.ajog.2020.05.017.

134. MCLAREN Jr. RA, LONDON V, ATALLAH F, MCCALLA S, HABERMAN S, FISHER N, STEIN JL, MINKOFF HL, Delivery For Respiratory Compromise Among Pregnant Women With COVID-19, Am J Obstet Gynecol (2020), doi: https://doi.org/10.1016/j.ajog.2020.05.035.

135. Buckley A, Bianco A, Stone J, Universal testing of patients and their support persons for COVID-19 when presenting for admission to Labor and Delivery within the Mount Sinai Health System, Am J Obstet Gynecol MFM (2020), doi: https://doi.org/10.1016/j.ajogmf.2020.100147.

136. Andrikopoulou, Maria; Madden, Nigel; Wen, Timothy; Aubey, Janice J; Aziz, Aleha; Baptiste, Caitlin D; Breslin, Noelle; D'Alton, Mary E; Fuchs, Karin; Goffman Dena; Gyamfi-Bannerman, Cynthia; Matseoane-Peterssen, Dara N; Miller, Russell S; Sheen, Jean-Ju; Simpson, Lynn L; Sutton, Desmond; Zork, Noelia; Friedman, Alexander M. Symptoms and Critical Illness Among Obstetric Patients With Coronavirus Disease 2019 (COVID-19) Infection, Obstet Gynecol May 27, 2020 – doi: 10.1097/AOG.0000000000003996

137. Hansen, Keith A; Stovall, Dale W. Ectopic Pregnancy During Coronavirus Disease 2019 (COVID-19), Obstet Gynecol: May 27, 2020 doi: 10.1097/AOG.0000000000003995

Note: Removed 1 June 2020 because the peer review correspondence makes clear that the case is hypothetical.

138. Ministério da Saúde, Secretaria de Vigilância em Saúde. Boletim Epidemiológico Especial da COVID-19 no Brasil. Número do boletim 17. 25 May 2020.

139. Sylvia M LaCourse, Alisa Kachikis, Michela Blain, LaVone E Simmons, James A Mays, Amber D Pattison, Carol C Salerno, Stephen A McCartney, Nicole M Kretzer, Rebecca Resnick, Rosemary L Shay, Leah M Savitsky, Anna C Curtin, Emily M Huebner, Kimberly K Ma, Shani Delaney, Carlos Delgado, Adrienne Schippers, Jeff Munson, Paul S Pottinger, Seth Cohen, Santiago Neme, Lori Bourassa, Andrew Bryan, Alex Greninger, Keith R Jerome, Alison C Roxby, Erica Lokken, Edith Cheng, Kristina M Adams Waldorf, Jane Hitti. Low prevalence of SARS-CoV-2 among pregnant and postpartum patients with universal screening in Seattle, Washington. Clinical Infectious Diseases ciaa675, https://doi.org/10.1093/cid/ciaa675

140. Mendoza, M., Garcia-Ruiz, I., Maiz, N., Rodo, C., Garcia-Manau, P., Serrano, B., Lopez-Martinez, R.M., Balcells, J., Fernandez-Hidalgo, N., Carreras, E. and Suy, A. (2020). Preeclampsia-like syndrome induced by severe COVID-19: a prospective observational study. BJOG. doi:10.1111/1471-0528.16339

141. NethOSS registry. 22nd May update

Appendix S2. Systematic review search strategy.

(("parturition"[MeSH Terms] OR "parturition"[All Fields] OR "birth"[All Fields]) AND ("pregnancy"[MeSH Terms] OR "pregnancy"[All Fields])) AND (((("severe acute respiratory syndrome coronavirus 2"[Supplementary Concept] OR "severe acute respiratory syndrome coronavirus 2"[All Fields] OR "sars cov 2"[All Fields]) OR ("COVID-19"[All Fields] OR "COVID-2019"[All Fields] OR "severe acute respiratory syndrome coronavirus 2"[Supplementary Concept] OR "severe acute respiratory syndrome coronavirus 2"[All Fields] OR "2019-nCoV"[All Fields] OR "SARS-CoV-2"[All Fields] OR "2019nCoV"[All Fields] OR (("Wuhan"[All Fields] AND ("coronavirus"[MeSH Terms] OR "coronavirus"[All Fields])) AND (2019/12[PDAT] OR 2020[PDAT])))) OR ("coronavirus"[MeSH Terms] OR "coronavirus"[All Fields])) OR (("coronavirus"[MeSH Terms] OR "coronavirus"[All Fields]) AND ("COVID-19"[All Fields] OR "COVID-2019"[All Fields] OR "severe acute respiratory syndrome coronavirus 2"[Supplementary Concept] OR "severe acute respiratory syndrome coronavirus 2"[All Fields] OR "2019-nCoV"[All Fields] OR "SARS-CoV-2"[All Fields] OR "2019nCoV"[All Fields] OR (("Wuhan"[All Fields] AND ("coronavirus"[MeSH Terms] OR "coronavirus"[All Fields])) AND (2019/12[PDAT] OR 2020[PDAT])))))

Appendix S3. Protocol for systematic review entitled 'Clinical factors affecting maternal transmission of SARS-COV-2 to the neonate: A systematic review avoiding duplicate cases'.

Authors: Kate F Walker, Keelin O'Donoghue, Jim G Thornton

Prepared after data collection had started. 13 May 2020

Summary

Objective

To estimate the risk of the neonate becoming infected with SARS-COV-2 by mode of delivery, type of infant feeding and mother-infant interaction

Design

A systematic review

Population

Women with COVID-19 in pregnancy.

Methods

A literature search using the following search strategy.

JGT searched PubMed only as follows

((("parturition"[MeSH Terms] OR "parturition"[All Fields] OR "birth"[All Fields]) OR ("pregnancy"[MeSH Terms] OR "pregnancy"[All Fields])) AND ("COVID-19"[All Fields] OR "COVID-2019"[All Fields] OR "severe acute respiratory syndrome coronavirus 2"[Supplementary Concept] OR "severe acute respiratory syndrome coronavirus 2"[All Fields] OR "2019-nCoV"[All Fields] OR "SARS-CoV-2"[All Fields] OR "2019nCoV"[All Fields] OR (("Wuhan"[All Fields] AND ("coronavirus"[MeSH Terms] OR "coronavirus"[All Fields])) AND (2019/12[PDAT] OR 2020[PDAT])))) OR ("coronavirus"[MeSH Terms] OR "coronavirus"[All Fields])

KFW search strategy

(("parturition"[MeSH Terms] OR "parturition"[All Fields] OR "birth"[All Fields]) AND ("pregnancy"[MeSH Terms] OR "pregnancy"[All Fields])) AND (((("severe acute respiratory syndrome coronavirus 2"[Supplementary Concept] OR "severe acute respiratory syndrome coronavirus 2"[All Fields] OR "sars cov 2"[All Fields]) OR ("COVID-19"[All Fields] OR "COVID-2019"[All Fields] OR "severe acute respiratory syndrome coronavirus 2"[Supplementary Concept] OR "severe acute respiratory syndrome coronavirus 2"[All Fields] OR "2019-nCoV"[All Fields] OR "SARS-CoV-2"[All Fields] OR "2019nCoV"[All Fields] OR (("Wuhan"[All Fields] AND ("coronavirus"[MeSH Terms] OR "coronavirus"[All Fields])) AND (2019/12[PDAT] OR 2020[PDAT])))) OR ("coronavirus"[MeSH Terms] OR "coronavirus"[All Fields])) OR (("coronavirus"[MeSH Terms] OR "coronavirus"[All Fields]) AND ("COVID-19"[All Fields] OR "COVID-2019"[All Fields] OR "severe acute respiratory syndrome coronavirus 2"[Supplementary Concept] OR "severe acute respiratory syndrome coronavirus 2"[All Fields] OR "2019-nCoV"[All Fields] OR "SARS-CoV-2"[All Fields] OR "2019nCoV"[All Fields] OR (("Wuhan"[All Fields] AND ("coronavirus"[MeSH Terms] OR "coronavirus"[All Fields])) AND (2019/12[PDAT] OR 2020[PDAT])))))

This will be supplemented by personal communication.

Possible duplication of cases.

Suspected duplication or overlap of cases in multiple reports will be dealt with conservatively.

Firstly we will identify reports including patients from the same hospitals using the GRID.ac research institution disambiguator. Unless there is clear internal evidence in the relevant papers that the cases do not overlap, we will assume that they do. In that case we will only include data from the largest or most informative report as seems appropriate.

Data will be extracted using a standardised form on mode of delivery, neonate infection status, infant feeding, mother-infant interaction.

For reported cases of neonatal infection, detailed information will be extracted on virology investigations and results

Intervention variables

Mode of delivery - vaginal or caesarean or not reported.

Infant feeding – breast, formula, expressed breast milk, or not reported.

Infant separation – isolated from mother, roomed in, or not reported.

Neonate alive or died

Neonate infected, or not infected. Using the criteria developed by Shah (15).

For reported cases of neonatal infection, detailed information will be extracted on virology investigations, both PCR and immunological, the sources from which samples were collected, their timing and their results.

Risk of bias assessment

For these observational studies we will judge risk of bias in two ways

a) In terms of how representative each paper is of the following three populations.

High representativeness = low risk of bias and vice versa.

1. The whole population of pregnant patients with SARS – Cov-2

2. The whole population of pregnant patients with COVID-19 i.e. symptomatic

3. Whole population of pregnant patients admitted to hospital with COVID -19

So, for example, a case report will be high risk of bias for all three populations.

A hospital based series, if it reports all cases, will be low risk of bias for 3, but high risk for 1 and 2.

UKOSS will be low risk of bias for 3. But high risk for 1 and 2.

b) For those subset of papers in which we had some baby data to assess the rate of infection after vaginal v CS, BF v bottle, or rooming in v isolated

We measure the bias in terms of proportion of babies tested in each of the above groups.

If all babies were tested in same way in both group = low risk of bias

If not = high risk

If no data U

Paper	Bias 1 All pregnant SARS-Cov2	Bias 2 all pregnant covid-19	Bias 3 all pregnant covid-19 in hospital	Baby testing bias	
	H, L or U	H, L or U	H, L or U	H, L or U	
