# Supplementary figures and images for: Maternal transmission of SARS‐COV‐2 to the neonate, and possible routes for such transmission: a systematic review and critical analysis
Source: BJOG. 2020 Jul 22;127(11):1324–36. doi: 10.1111/1471-0528.16362 (PMC7323034; doi:10.1111/1471-0528.16362)

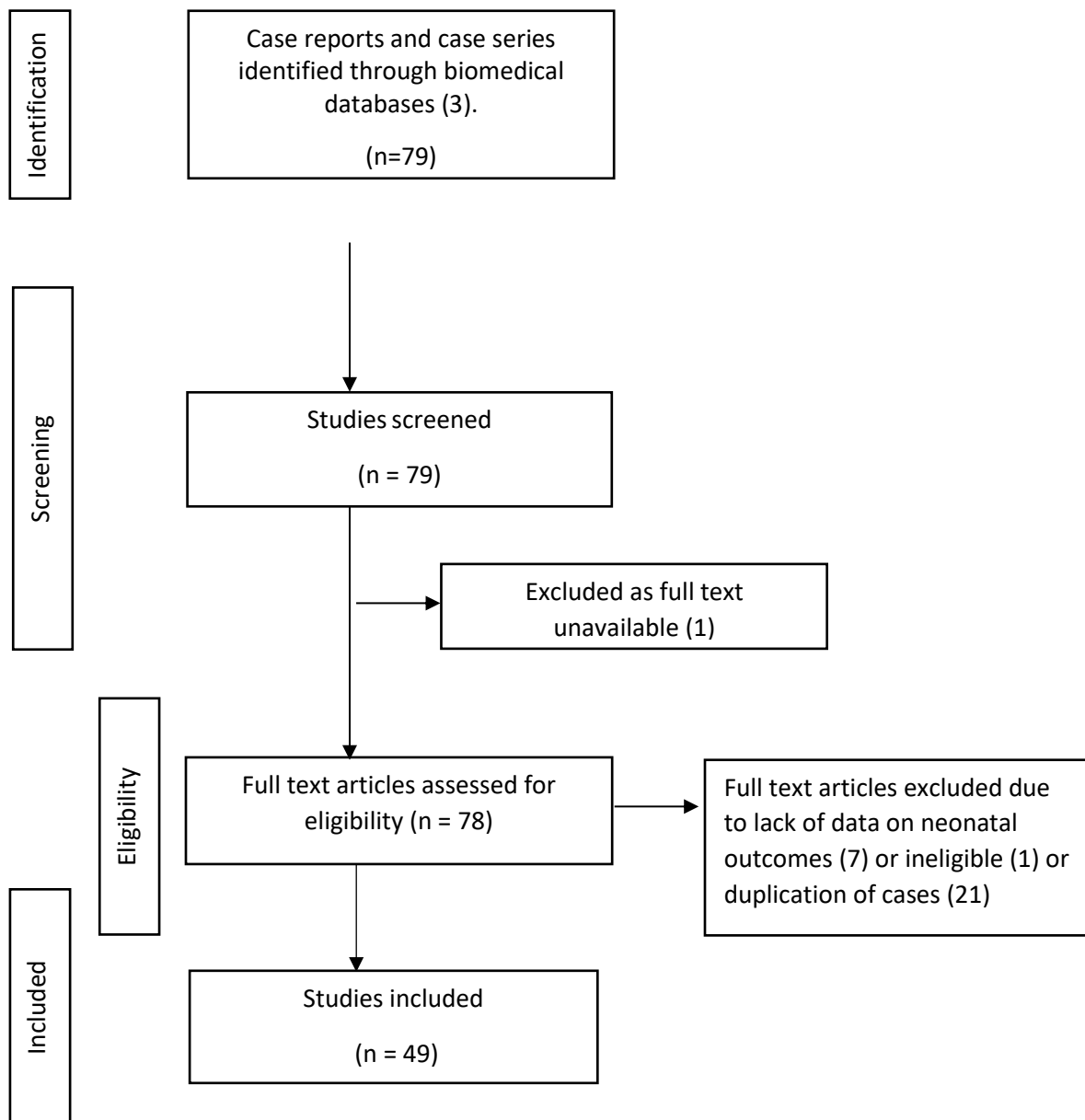

**Figure S1.** Study flow chart.

Supplement: Supplementary file 2 — Figure S1. Study flow chart. [file BJO-127-1324-s009.pdf]
