# Supplementary material for: Maternal transmission of SARS‐COV‐2 to the neonate, and possible routes for such transmission: a systematic review and critical analysis
Source: BJOG. 2020 Jul 22;127(11):1324–36. doi: 10.1111/1471-0528.16362 (PMC7323034; doi:10.1111/1471-0528.16362)
Supplement: Supplementary file 3 — Table S1. Virology results and summary of cases for babies with possible vertical transmission. Table S2. Risk of bias assessment. [file BJO-127-1324-s008.pdf]

Table S1. Virology results and summary of cases for babies with possible vertical transmission

	Case 1	Case 2	Case 3	Case 4	Case 5	Case 6	Case 7	Case 8	Case 9	Cases 10-21	Case 22	Case 23	Case 24	Case 25-28
**Study (Appendix A)**	8	8	8	15	51	67	70	76	79	92	100	103	111	117
**Mode of delivery**	CS	CS	CS	CS	CS	CS	CS	CS	NVD	4 NVD<br>8 CS	CS	CS	CS	3 NVD<br>1 CS
**Gestational age at delivery**	40	40	31	39	33	30	32	37	37	Not reporter for individual cases	26	35	27	Not reported
**Birthweight**	3250	3360	1580	3250	2970	Not reported	2350	3390	3120	Not reporter for individual cases	970	2930		Not reported
**Details of mother-infant interaction post delivery**	Strict infection prevention and control Procedures were implemented during the delivery. No further details	Strict infection prevention and control procedures were implemented during the delivery. No further details	Strict infection prevention and control procedures were implemented during the delivery. No further details	Not reported	Immediately separated. No delayed cord clamping or skin to skin. Intubated in another room	Immediately separated	Immediately separated	Immediately separated until D5. Returned home with mother on D5.	Immediately separated	Not reported	Immediately separated from mother, but skin to skin contact on D6 prior to positive swab result in mother	Immediately separated before cleansing and swabs taken, then returned to mother	Immediately separated	No separation, rooming in
**Infant feeding**	Not reported	Not reported	Not reported	Not reported	Formula fed	Formula fed	Formula fed	Formula fed from D0-D5. Mother breast fed from D5 while wearing a mask	Not reported	Not reported	Expressed breast milk until positive diagnosis in mother	Breast fed with a surgical mask	Not reported	Breast feeding
**Infant condition**	D2 infant experienced lethargy and fever, with unremarkable physical examination results, moved to NICU. CXR showed pneumonia	Presented with lethargy, vomiting, and fever. A physical examination was unremarkable. CXR showed pneumonia	Resuscitation was required. The infant's Apgar scores were 3, 4, and 5 at 1, 5, and 10 minutes after birth. Neonatal respiratory distress syndrome and pneumonia confirmed by chest radiographic image on admission resolved on day 14 of life after treatment with non-invasive ventilation, caffeine, and antibiotics. He also had suspected sepsis, with an Enterobacter agglomerates–positive blood culture,	Mild shortness of breath. Chest x-ray revealed mild pulmonary infection. The shortness of breath relieved quickly under neonatal care and monitoring. The neonate was discharged after two weeks	Apgar's scores of 6 and 8 at 1 and 5minutes. Required intubation due to general depressed state. Required ventilatory support for 12 hours, then extubated and placed on continuous positive airway pressure, with favourable outcome and not requiring antibiotic treatment. D6	Intubated for prematurity. Developed pneumonia D2. Remains intubated and stable	Apgar scores of 8 and 9 at 1 and 5 minutes. Required antibiotics for pyrexia.	Normal Apgars, asymptomatic, remained well	Normal Apgars, asymptomatic, remained well	Not reporter for individual cases	Complications of prematurity but no signs of infection	Apgar scores were 9 at 1 minute and 9 at 5 minutes. Required admission to NICU at 36 hours for hypoglycaemia and feeding difficulites. Empiracally treated with antibiotics, negative septic screen. Discharged day 4	Apgar scores of 1, 3 and 6 at 1, 5 and 10 min respectively. No infectious symptoms	None had respiratory symptoms

				leukocytosis, thrombocytopeni a and coagulopathy, which improved with antibiotic treatment.		presented mild respiratory difficulty and sporadic cough requiring supplement al oxygen with nasal cannula. Imaging and laboratory testing remain normal.									
	**Specimen**														
Materna l	Vaginal swab									DO Negative		D1 positive			
	Amniotic fluid	Negative	Negative	Negative				Positive	Negative						
	Nasopharynge al swab	Positive	Positive	Positive	Positive antenatally , day not specified	Positive D1 postnatally	Positive antenatally, two days prior to delivery	Positive antenatally, three days prior to delivery	Positive antenatally, seven days prior to delivery	D0 Positive		Positive two days prior to birth and D7	Positive six days prior to delivery	Timing not specified	
	Sputum														
	Blood					D1 postpartum maternal serology negative IgG and IgM levels D4 postpartum positive IgG and IgM (D9 after symptom onset)									
	Faeces									D0 Positive					
	Breast milk	Negative	Negative	Negative					Negative	D0 Negative		D2 positive			
	Placenta								Negative	D0 Negative		D0 positive			
Neonata l	Blood					D0 negative IgM and IgG			D1 negative IgM, slightly positive IgG			D2 negative, positive D4			
	Urine														
	Umbilical cord	Negative	Negative	Negative				Negative	Negative	D0 negative IgM, positive IgG					
	Nasopharynge al	Positive D2 and D4	Positive D2 and D4	Positive D2 and D4	Positive D3	Positive D1 and D3	Negative D1 Positive D7	Negative D0 Positive D1	Negative D1 and D3 Positive D15	D0 positive D1 negative	6/12 neonates positive D0 6/12 neonates positive > 12 hours after birth	D0, D2 and D7 positive	D3 positive		
	Anal/rectal	Positive D2 and D4	Positive D2 and D4	Positive D2 and D4											

Table S2. Risk of bias assessment

**Paper**	**Appendix A STUDY NO.**	**Bias 1** All pregnant patients with SARS-CoV2	**Bias 2** All pregnant patients with COVID-19 infection (symptomatic)	**Bias 3** All pregnant patients with COVID-19 admitted to / in (a/any) hospital	**Baby testing bias**
		*H, L or U	*H, L or U	*H, L or U	*H, L or U

***H (high); L (Low); U (no data)**

China

Zongnan Hospital of Wuhan University grid.413247.7	1	H	H	H	U
Wuhan Children's Hospital<br><br>Maternal and Child Hospital of Hubei Province	8, 30	H	H	L (neonates)<br><br>H (mothers; unclear)	L
Union Hospital, Tongji Medical College, Huazhong University of Science and Technology grid.412839.5	2a, 6	H	H	L (noting duplicate reports)	H (2a)
Tongji Hospital, Tongji Medical College, Huazhong University of Science and Technology grid.412793.a	15	H	H	L	H
First Affiliated Hospital of Sun Yat-sen University	3	H	H	H (too many hospitals)	H
Renmin Hospital of Wuhan University grid.412632.0	36, 37	H	H	H (impossible - due to overlapping reports)	H
Affiliated Infectious Hospital of Soochow University, Suzhou.	19	H	H	H	H (one case)
Beijing YouAn Hospital	34	H	H	H	H (one case)
No. 2 People's Hospital of Hefei City Affiliated to Anhui Medical University	62	H	H	H	H (one case)

USA

New York Presbyterian & Columbia	27 (+14)	H	H	L	L
MedStar Washington Hospital Center	21	H	H	H	H (one case)
Good Samaritan hospital, Ohio	50	H	H	H	H (one case)
Hospital of the University of Pennsylvania	65	H	H	H (series/ only critically ill)	U
Washington University in St. Louis, Missouri	69	H	H	H	H
New York Winthrop Hospital	91	H	H	H	H (one case)
New York University, Langone Health	85	H	H	L	H

San Francisco	89	H	H	H	H
Livingstone, New Jersey	111	H	H	H	H (twins)
Stanford University Hospital	115	H	H	H	H (one case)
Weill Cornell Medicine, New York	118	H	H	H	H (one case)
Michigan	123	H	H	L	L
Maimonides Medical Center, Brooklyn	113	H	H	L	L

Honduras

Hospital Escuela of Tegucigalpa	18	H	H	H	H (one case)

Sweden

Southern General Hospital, Stockholm	20	H	H	H	H (one case)

Korea

Daegu Fatimal Hospital	22	H	H	H	H (one case)

Turkey

Ankara University	31	H	H	H	H (one case)

Italy

IRCCS Fondazione Policlinico Universitario Agostino Gemelli, Rome	76	H	H	L	H (2 cases)
Sant'Anna Hospital, Turin	79	H	H	H	H (one case)
Parma	109	H	H	H	H
Italian hospitals	117	H	H	L	L

Portugal

Hospital Pedro Hispano in Porto (105)	105	H	H	L	L

Australia

Gold Coast University Hospital	45	H	H	H	H (one case)

Canada

Mount Sinai Hospital Study	48A	H	H	H	U

France

Antoine Béclère hospital Study 48B	48B	H	H	H	U

Spain

Madrid	125	H	H	L	L

Peru

British American Hospital Study 51	51	H	H	H	H (one case)

India

Designated COVID hospital	58	H	H	H	H (one case)

Iran

Vali-e-asr Hospital, Zanjan	43	H	H	H	U
Tehran/Rasht/Qom/Zanjan	67	H	H	H	H
Imam Khomeini Hospital, Sari	70	H	H	H	H (one case)
Imam Reza hospital of Tabriz	101	H	H	H	H (one case)

UK

UKOSS	92	H	H	L	H

Belgium

Cliniques Universitaires Saint Luc	100	H	H	H	H

Netherlands

NethOSS	141	H	H	L	H
